# An integrated analysis of naval platform survivability for mission resilience

**DOI:** 10.1038/s41598-025-29698-5

**Published:** 2025-12-04

**Authors:** Anthony Woolley, Alexander Gargano, Serap Aksu, Grant Gamble, Daniel Foos

**Affiliations:** 1 Platforms Division, Defence Science & Technology Group, 506 Lorimer Street, Fishermans Bend, Victoria, 3207 Australia; 2 Test and Evaluation Solutions LLC, Warrenton, USA

**Keywords:** Integrated survivability, Survivability, Vulnerability, Recoverability, Damage control, Warship, Computational science, Information theory and computation, Computational methods, Mechanical engineering

## Abstract

Mission resilience is a primary consideration for naval platforms operating in contested maritime environments. It is a concept that encapsulates a platform’s ability to endure, and to survive threats that affect the achievement of mission objectives. Historically, platform survivability analysis was constrained by “stove piping” the analysis within individual survivability domains (susceptibility, vulnerability and recoverability). However, the trade-space combining susceptibility, vulnerability and recoverability in a temporal framework enables holistic survivability analysis. This is referred to as “Integrated Survivability”. To demonstrate the utility of Integrated Survivability analysis, and its contribution to mission resilience, a naval platform survivability case study was developed. The case study examined the achievement of a mission objective for four platform configurations exposed to a detonating weapon threat. There were three primary outcomes. Firstly, the case study demonstrated a platform general arrangement configuration change to improve crew access, instead of a technological solution, provided the best outcome for achieving the mission objective. Secondly, the case study demonstrated the use of a warship computer model specifically designed for naval platform survivability analysis. This warship model is an unclassified, generalised naval platform representation designed to foster collaboration and for documenting research in the open literature. Finally, the case study enabled the development of a workflow to facilitate Integrated Survivability analysis for the enhancement of the fleet-in-being and assessment of future naval platform acquisitions.

## Introduction

Naval platform Integrated Survivability analysis provides insights into whole-of-platform and mission survivability. It is an assessment of platform susceptibility, vulnerability and recoverability in the temporal domain to examine platform resilience to (combat and non-combat) threat encounters^1,2^. The outcomes contribute toward naval capability acquisition and provide assurance of mission readiness for the fleet-in-being. During naval capability acquisition, Integrated Survivability analysis facilitates assessment of competing platform designs against threat environments to which the platform will be exposed. This enables capability trade-off to support mission resilience by comparing (for example) operational tactics, system redundancy, zoning, separation, fire safety systems, fire insulation, armour plating, and/or recoverability procedures to optimise the combination of survivability control measures afforded to the platform. For the fleet-in-being, the consequence of technology insertion can be examined by performing a cost–benefit analysis of the proposed new technology comparing it to an increase (or decrease) in platform and mission survivability. Integrated Survivability analysis will also inform the decision-making process for mission planning to assist with the identification of a suitable platform for the achievement of the mission requirement. Ultimately, the aim of Integrated Survivability analysis is to maximise the probability of mission success while minimising the platform’s risk of exposure to the threat environment. Minimising the risk of exposure includes, maximising the ability to avoid a threat; minimising the damage inflicted by the threat; and maximising the ability to recover mission capability after damage has occurred. Noting, it is not possible to completely eliminate that risk of exposure.

Documented in the literature are examples of platform survivability analysis for specific survivability domains. For example, Friebe et al.^3^ demonstrated a methodology for analysing the relationship between systems and components to minimise platform vulnerability. Liwang and Jonsson^4^ derived measures of survivability in the vulnerability domain, calculating the probability of kill, *P*_*k*_, for various platform functions. They presented examples using three generic frigate configurations against two threat types. The aim was to demonstrate the effects of different threat types against platform configurations; and highlight the effects of system redundancy and separation in relation to vulnerability. Piperakis and Andrews^5^ performed a survivability analysis across the susceptibility, vulnerability and recoverability domains. However, they recognised the temporal nature of the recoverability domain and a need for human factors analysis. Consequently, they derived weighted performance measures for the recovery of various platform functions. They presented survivability as a Star Plot, with calculated platform susceptibility, vulnerability and recoverability measures forming the apex of each corner on the Star Plot. This was performed for three generic platforms: a corvette; a destroyer; and an oil replenishment platform.

Analysis tools, such as the *Integrated Recoverability Model* (*IRM*)^6^, *Purple Fire*^7,8^, the demonstrated use of *SURVIVE* with platform signature modelling software^9^, and the development of the *Naval Damage Integrated Recoverability Toolset* (*NavDIRecT*)^2^ exemplify the integration of survivability analysis. However, these tools demonstrated limited aspects of Integrated Survivability, such as: vulnerability (fire and/or flooding)-recoverability; susceptibility (radar cross section)-vulnerability; and vulnerability (blast)-recoverability. Currently, there is no whole-of-domain Integrated Survivability modelling and simulation (M&S) capability.

Earlier publications highlighted the importance of incorporating the three survivability domains into platform and mission survivability design. For example, Naval Sea Systems Command (NAVSEA) developed the engineering management discipline, Total Ship Survivability (TSS)^10^. TSS integrated platform survivability into all phases of the design process to ensure delivery of platforms capable of fulfilling the capability requirements (and, consequently, fulfilling the mission requirements). Later, the Office of the Chief of Naval Operations Instruction (OPNAVINST) 9070.1B^11^, defined survivability as “a measure of both the capability of the ship, mission critical systems, and crew to perform assigned warfare missions, and of the protection provided to the crew to prevent serious injury or death.” Woolley and Whitehouse^1^ then provided a definition for Integrated Survivability to distinguish it from the generalised term, survivability. They also presented a framework for Integrated Survivability analysis, without practical demonstration of the benefits afforded by such a framework. Furthermore, a methodology to perform Integrated Survivability analysis was not discussed. Consequently, an applied engineering research case study was proposed to examine and identify the requirements for such a methodology. The case study advances interdisciplinary design and operational integration in the shipbuilding, survivability and operational mission planning communities. The impetus for the work supports the Royal Australian Navy’s (RAN’s) objective that “maximises the likelihood of achieving the specified operational effect for the defined tasking”^12^.

The case study utilised a generic warship computer aided design (CAD) model^13,14^. The generic warship model is an unclassified naval platform representation that uses historic naval platform design principles. The model was designed for use in collaborations and to report findings in the literature where sharing classified information is prohibited. Utilising the generic warship model, a scenario was created such that four configurations of the generic warship were exposed to an airborne detonating weapon threat. The aim of the analysis was to recommend an optimal platform configuration demonstrating mission resilience against the threat encounter.

The design of the case study supported several goals beyond the need to demonstrate the application of Integrated Survivability analysis. The case study facilitated understanding of the Integrated Survivability M&S process; identified data and M&S requirements to perform Integrated Survivability analysis; and demonstrated the use of the generic warship model and identified deficiencies within that model. A primary output was the creation of a workflow to guide future naval platform Integrated Survivability analysis. The development of the scenario and performing the analysis demonstrating the role of Integrated Survivability analysis for naval platform mission resilience is presented. The resultant methodology is discussed and presented in the form of a tabulated workflow.

This paper advances prior work presented by Woolley et al.^1,2,13^. The prior work discusses the need for Integrated Survivability analysis, a proposed framework for Integrated Survivability analysis and its functional requirements, and the ability to quantify platform survivability.

## Integrated survivability

**Fig. 1:**
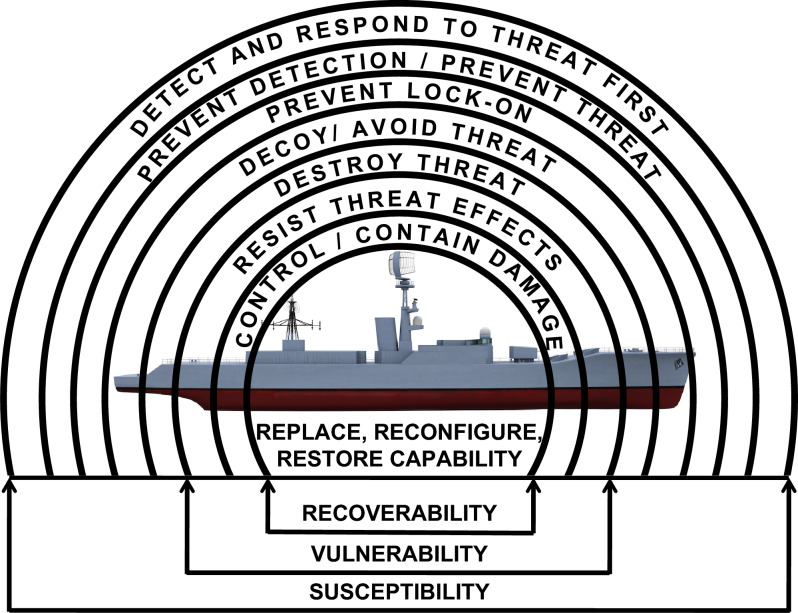
Integrated Survivability Onion conceptualising the layers of defence afforded to naval platforms.

Woolley and Whitehouse^1^ presented a detailed description of Integrated Survivability, and an overview will be presented here. To understand naval platform Integrated Survivability, it is important to consider the domains contributing to a survivable platform: susceptibility; vulnerability; and recoverability. These domains provide tactical, electronic and physical barriers against the (combat and non-combat) threat environment. The Integrated Survivability Onion, presented in Fig. 1, is a common depiction of each domain in naval platform survivability. The layers within the Integrated Survivability Onion form the resilient platform trade-space, with consideration to the operational profiles to which the platform may be exposed (including task force and joint operations). The trade space consists of the tactical procedures and the technology afforded to the platform: to avoid and/or destroy a threat (susceptibility); to resist the consequences of threat effectors (vulnerability); and to recover capability to enable continuation of the mission and/or to ensure the safety of the crew and/or platform (recoverability).

Changes to susceptibility, vulnerability and recoverability in a platform’s configuration, tactical procedures and/or crew standard operating procedures (SOPs) may affect the platform’s ability to avoid and/or destroy a threat, resist threat effects, and/or recover platform capability. However, due to the need for human factors analysis and the temporal nature of survivability analysis, recoverability operations (to recover system functionality and platform capability) are sometimes not considered. Recoverability operations are informed by Command Aims, which are informed by the evolving damage situation, the external threat environment, and mission objectives. Consequently, Command Aims can change as a result of a threat encounter and during recoverability operations.

The evolving damage situation, the ongoing threat environment, and the effect of recoverability operations also affect the state of the platform. Command Aims and platform state (such as, residual mission capability and ongoing damage) in relation to achieving a mission objective have not previously been considered in survivability analysis documented in the literature. Therefore, this was a primary motivation to perform the survivability case study presented herein. Hence, the case study included demonstration of the effects of crew recoverability operations (including, damage control) in support of a Command Aim to achieve a mission objective.

### Mission resilience and integrated survivability

Many key words and phrases are used in doctrine, policy, and scientific publications when describing naval platforms, survivability, and the threat environment. These include, “float, move, fight”, “fight and survive”, “high threat environment”, “resilience”, “endurance”, “survivability”, “Integrated Survivability”, “operational effect”, “operating and support intent”, “battle ready platform” and “seaworthiness”. Usage of the terminology requires in depth familiarity and understanding of the defence system. Consequently, in the open literature, simplifications are utilised. Simplifications such as, “mission requirement” defined to be the "the role a platform is required to fulfil"; and “mission objectives” defined as “the goals to achieve in fulfilment of a mission requirement”. Mission resilience is the platform’s ongoing ability to achieve the mission objectives, consisting of:

- platform survivability to the threat environment;
- platform system redundancy and flexibility;
- ability for continuous operation;
- adaptability to the evolving threat environment; and,
- crew skill and safety.

A platform fulfils mission objectives via the application of “capabilities” afforded to the platform. Capabilities are comprised of the systems installed on the platform. Each system has a functionality and many systems will contribute to form one capability; and each system can contribute to multiple capabilities. Furthermore, systems are not just hardware and software. Systems also include the crew. A naval platform is a sociotechnical environment. That is, the crew, their skills, their behaviour and their interaction with other platform systems.

The requirements phase of naval platform acquisition will identify mission requirements the platform is to fulfil. For example, a platform might be required for anti-submarine warfare. Secondary requirements might be placed on the platform to support other warfare roles (for example, anti-aircraft warfare). Within each of these requirements, objectives will be specified, such as: the time-on-station (that is, endurance); and the ability, and timeframe in which, to recover from damage events.

Platform upgrades are required due to capability gaps (resulting from a changing mission requirement, and a changing threat environment); equipment and system technology refresh cycles; and disruptive technology. Integrated Survivability analysis will contribute to understanding the capability gap, and supports identification of suitable options to fill that gap.

Prior to deployment, specifics of the platform’s mission requirement(s) are identified. These are the mission objectives. In combat environments, weapon threats are also identified. Simulating the mission and performing Integrated Survivability analysis will: assist the crew’s understanding of their mission objectives and mission requirement; make them aware of the consequences of threat encounters; and enable them to become mission ready.

Consequently, to understand mission resilience, there is a need to understand platform systems and the contribution to platform capability; and there is a need to understand platform capabilities contributing to individual mission objectives and the overall mission requirement. There is a need to understand the remaining capability after a threat encounter and the effect on platform performance for the achievement of the mission requirement.

Integrated Survivability analysis provides for whole-of-platform survivability across an entire scenario. Monte Carlo simulation will be utilised to examine variations within a scenario and explore multiple scenarios using off-the-shelf and bespoke M&S software on an “as required” basis^1^. Integrated Survivability analysis will quantify naval platform survivability against multiple threats for multiple scenarios to enable trade-off analysis across, and within, the domains of susceptibility, vulnerability and recoverability. Further trade-off analysis will be required against other platform design parameters to maintain, for example, weight margins, power load, speed in the water, and endurance. For example, including armour plating around critical systems will minimise *P*_*k*_ against fragmentation impact but the armour plating will affect weight margins. Increasing the crew size may improve damage control and recoverability operations but it will affect the provision of victualing and, therefore, platform endurance. Increasing system redundancy will increase mission resilience but it will affect weight margins, power load, and system layout configuration. These are the types of considerations to be examined when designing a new platform or upgrading an existing platform. A formalised Integrated Survivability analysis methodology will enhance the trade-off process to maximise mission resilience.

## Defining the case study

This case study is the first to be performed demonstrating the process of integrating available M&S capability to support Integrated Survivability analysis for the RAN. However, survivability analysis in the individual domains of susceptibility and vulnerability has previously occurred in support of RAN inquiries (for example, investigating the 1941 wartime loss of HMAS *Sydney* II^15^; and investigating the 1998 fire onboard HMAS *Westralia*^16^). Defining the case study followed considerations for design threat scenarios in Integrated Survivability analysis presented by Woolley and Whitehouse^1^. The following sections define: case study objectives; assumptions; constraints; and M&S tools utilised to perform the case study. The scenario is also defined, including a description: of the generic warship model; the threat to which the platform is exposed; and platform configurations examined.

### Case study objectives

Three objectives were to be achieved performing the case study:

1. to demonstrate the utility of Integrated Survivability analysis was the primary goal of the exercise. This was achieved via an applied Integrated Survivability analysis example utilising a formal scientific process of identifying options, hypothesising solutions, investigation and testing, and documenting results; and, proving the applicable benefits to the platform design and the enhancement of mission resilience while providing confidence to stakeholders to perform similar, future, analyses;
2. to understand Integrated Survivability modelling and data requirements. The aim was to identify the data and data formats required by the survivability M&S tools. This is an important consideration due to the M&S tools each requiring data in formats that may not be compatible with other survivability M&S tools. There is a need to share data between the M&S tools and, consequently, a need to format the data accordingly; and,
3. to develop an Integrated Survivability workflow. This was an important output. It is anticipated the workflow, combined with M&S and data requirements, will guide future Integrated Survivability analysis.

### The generic warship virtual platform model

**Fig. 2:**
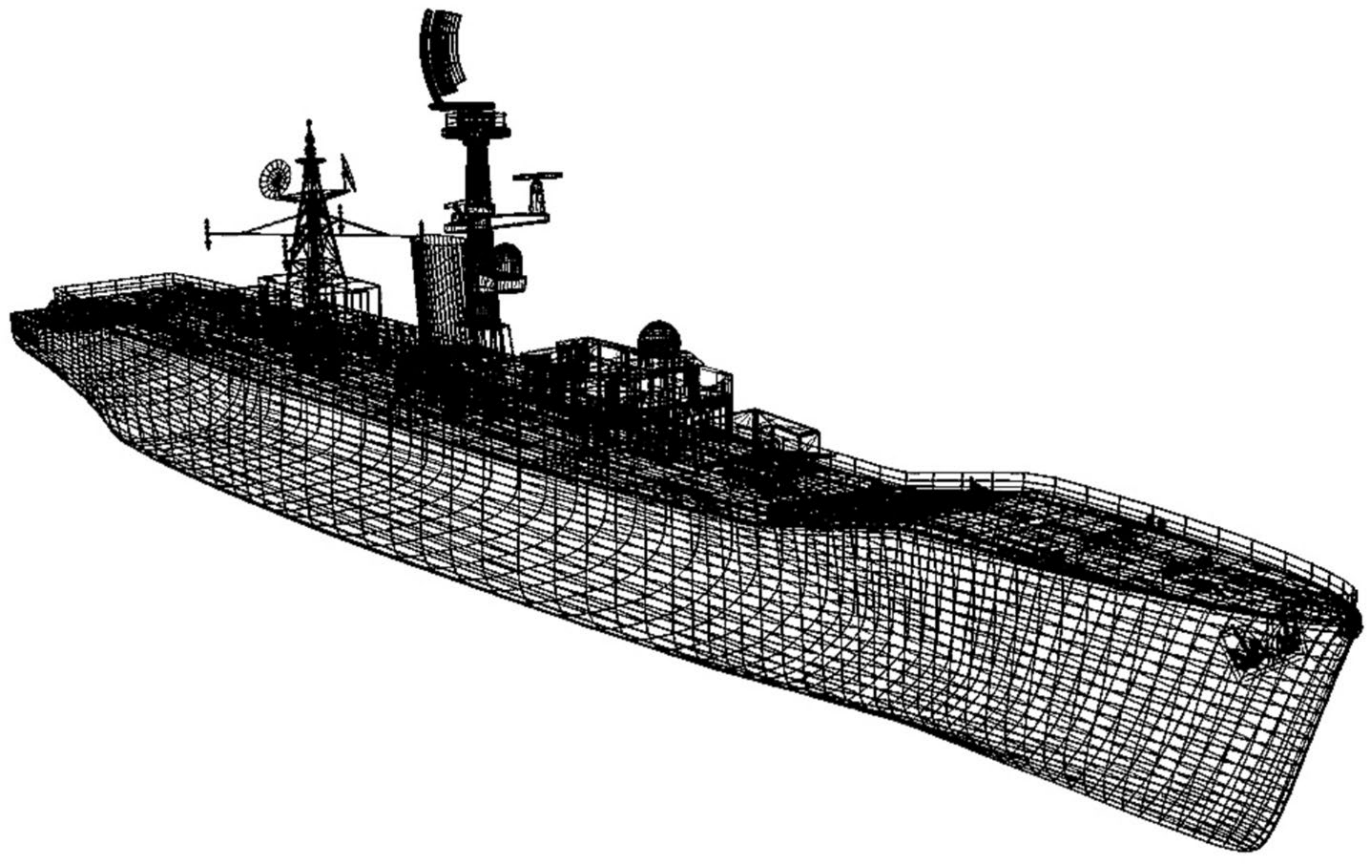
A three-dimensional, wire-frame representation of the virtual platform, VSRS *Arion*.

The generic warship model is referred to as the Virtual Survivability Research Ship (VSRS) *Arion*^13,14^. VSRS *Arion*, shown in wireframe in Fig. 2, is a virtual platform model developed as a research and analysis tool specifically for use in naval platform survivability studies. As previously documented^13,14^, the VSRS *Arion* model was created using Autodesk *Maya*^17^. The VSRS *Arion* imagery depicted in this case study was a direct output from the creation of the model. The model may be freely shared to facilitate collaboration with research institutions (nationally and internationally) for the development of naval platform survivability analysis capabilities. Prior to this case study, the VSRS *Arion* model had not been tested in any of the available naval platform survivability M&S tools. Consequently, the case study provided an ideal opportunity to perform such testing.

VSRS *Arion* was derived from the River class destroyer escort, HMAS *Derwent*. The River class destroyer escort served with the RAN and was an evolution of the Royal Navy Type 12 M Rothesay class frigate. HMAS *Derwent* was constructed at Williamstown Dockyard, Victoria, Australia, and commissioned in April 1964. During its service life, the primary weapons of HMAS *Derwent* included two 4.5" guns in a twin turret; a Seacat anti-aircraft missile system; the Australian designed Ikara anti-submarine missile system; and two triple mounted anti-submarine torpedo tubes. Crew complement consisted of approximately 240 officers and enlisted personnel. HMAS *Derwent* was decommissioned in 1994 and subsequently used in explosive, and fire and smoke trials known as the Ship Survivability Enhancement Program (SSEP)^19^. At the conclusion of the SSEP, HMAS *Derwent* was scuttled in deep water off the coast of Western Australia.

The naming of VSRS *Arion* was inspired by the motto of HMAS *Derwent*, “Swift and Deadly”, and the great, swift horse Arion of Greek mythology. VSRS *Arion* is also so named to distinguish it from HMAS *Derwent*, thereby avoiding confusion when discussing each platform.

Due to the age of the River class destroyer escort, it was deemed there would be no further security sensitivities associated with the class. However, the age of the class is also detrimental. The historic River class destroyer escort platform design philosophies do not reflect current design philosophies. Most of these issues can be overcome, if necessary, during the M&S scenario design phase, by specifying modern materials and equipment (*Modelling Assumptions* and *VSRS Arion Platform Configurations*).

**Fig. 3:**
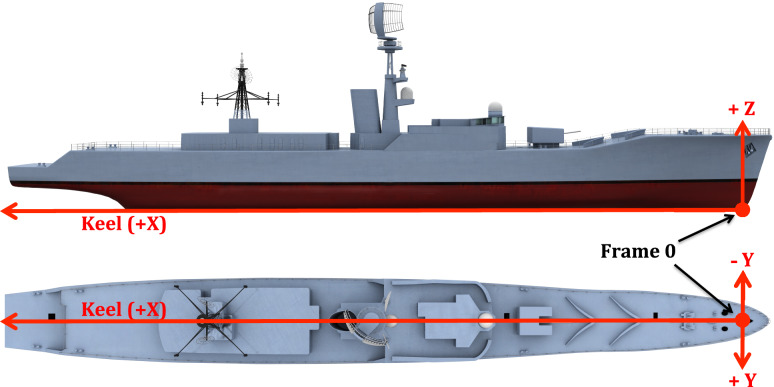
The global datum on the centerline at Frame 0.

**Fig. 4:**
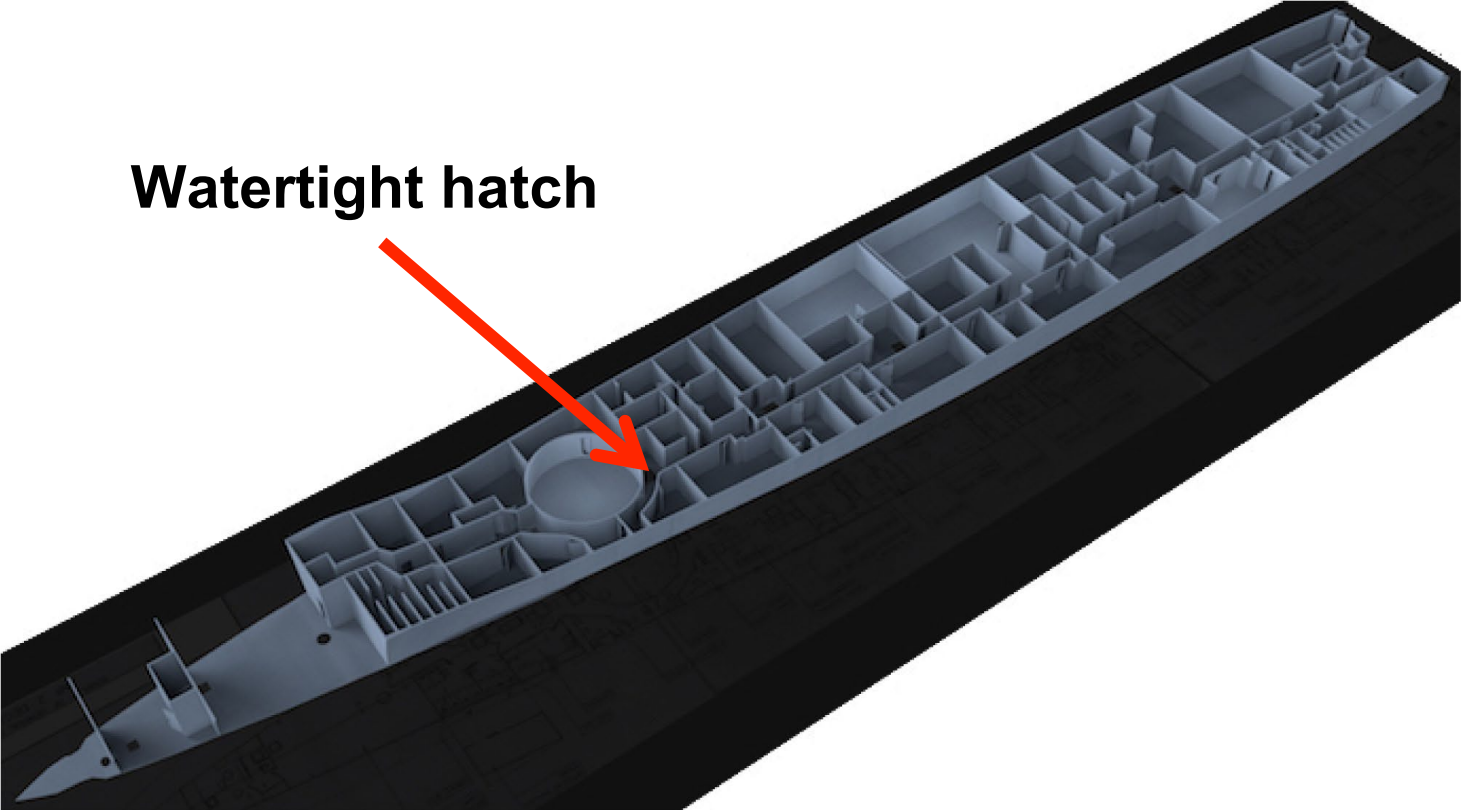
A watertight hatch in 2 Deck, located at (X, Y, Z) = (35.42, -0.53, 6.88) m.

Locating objects within VSRS *Arion* is with respect to a global datum measured from the bow of the platform^13^. Depicted in Fig. 3, the global datum is located on the keel-line at Frame 0. Objects within VSRS *Arion* have a positive X-axis value aft of Frame 0. Objects starboard of the keel-line have a positive Y-axis value; and objects to the port of the keel-line have a negative Y-axis value. All objects have a positive Z-axis value (since they are above the keel-line). The unit of measurement is metres (m). For example, consider a watertight hatch located in the deck of a passageway on 2 Deck. The hatch connects 2 Deck with 3 Deck. The approximate location of this hatch is shown in Fig. 4 and the location of the centroid for the hatch is (X, Y, Z) = (35.42, -0.53, 6.88) m.

### The weapon threat

The weapon used in the case study was a rocket-propelled grenade (RPG) containing two explosive charges. The inclusion of the second charge allowed the first charge to breach the hull, thereby providing a path for the second charge to penetrate the hull and detonate within the platform. The RPG is a shoulder-launched weapon, with a line-of-sight (“eyeball”) targeting system. The RPG specification defined for the case study is presented in Table 1.

**Table 1:** RPG specification used in the scenario.

Length	0.618 m
Diameter	0.072 m
Wingspan	0.218 m
Weight	1.48 kg
Warhead	0.36 kg high melting explosive (HMX)
Propulsion	Rocket
Maximum Speed	133 ms^-1^
Damage Radii	3.2 m

### Selection of survivability M&S tools

Four M&S tools contributing to naval platform survivability analysis were selected for use in the case study. They were: *Unified Vulnerability Assessment Model* (*UVAM*) (*UVAM* was previously referred to as *Vulnerability Lethality Capability Analysis* (*VuLCAn*)^1^); *Fire and Smoke Simulator* (*FSSIM*); *IRM*; and *Fire Dynamics Simulator* (*FDS*). Only *UVAM*, *IRM* and *FSSIM* contributed directly to analysing the scenario. *FDS* was selected to gain experience in the use of the software and further demonstrate utilisation of the VSRS *Arion* model. The use of *FDS* did identify an issue with the VSRS *Arion* model and this is discussed in Section "Identified M&S issues" *Identified M&S Issues*.

#### UVAM

*UVAM* is a bespoke air explosion M&S tool developed by Defence Science and Technology Group (DSTG). It is an evolution of the vulnerability assessment method software known as *CVAM*^20^. The output from *UVAM* is *P*_*k*_ for platform structure, components and systems. *UVAM* utilises empirical algorithms to model fragment penetration and blast spread from the weapon detonation. The *UVAM* algorithms used in the case study are presented in Section "Vulnerability: blast and fragmentation" *Vulnerability: Blast and Fragmentation*.

#### FSSIM

*FSSIM* is an off-the-shelf network fire and smoke M&S tool, specifically developed for use in naval platform survivability analysis^21^. *FSSIM* simulates a fire within a node in the network, where a node usually represents a room or a compartment. Models for heat and gas transfer via compartment boundaries and openings are also included. The network model is a simpler method than computational fluid dynamics (CFD) based models because there is only one set of values for each node. Conversely, a room volume in a CFD model is “meshed” (divided into many cells), resulting in many sets of values for each room. Consequently, *FSSIM* is capable of simulating fire scenarios quickly and is, therefore, ideal for integration into a naval platform survivability analysis capability.

#### IRM

*IRM* is a commercial-off-the-shelf agent-based network connectivity post damage temporal simulation developed to evaluate the complex interaction of crew actions combined with integrated shipboard system behaviours. *IRM* system connectivity and interaction is established via: data (such as, for valve control; combat command; and communications) and power cabling (DC24V, AC 50/60 Hz and 400 Hz); auxiliary and support pipe work (including, fuel oil; seawater cooling; fire main and drainage; freshwater cooling; and potable water); and ducting between relevant components (such as, propulsion and power intake/exhaust; venting; and heating, ventilation and air conditioning). Resource supply and consumer relationships are assigned to support the identification of platform design issues. Simulation of the shipboard system-of-systems with realistic interactive crew behaviours inclusive of training skill sets, variable pedestrian movement speeds, protective gear status, and other movement attributes provide for realistic evaluation of shipboard recoverability. Integrated and third-party software can be included to expand capabilities for flooding (not utilised in this case study) and fire and smoke spread (as applied with *FSSIM* in this case study).

#### FDS

*FDS* is an off-the-shelf CFD M&S tool to model the flow of fire and smoke, developed by the National Institute of Standards and Technology (NIST)^22,23^. *FDS* simulates the output of fire, including heat and combustion products, and the movement of smoke and air; and heat transfer via radiation, convection and conduction. *FDS* numerically solves a form of the Navier–Stokes equations^23^ for fluid and heat transport within mesh cells for the space and geometry defined by the user. Consequently, *FDS* can simulate complex fire scenarios, but this is computationally demanding due to the large number of calculations required.

The use of *FDS* did not contribute to all the case study aims. Experience with other fire and smoke M&S tools had previously been demonstrated in a naval platform survivability environmen^2,24^, but not *FDS*. Consequently, *FDS* was utilised to increase understanding of fire and smoke M&S tools by providing the analysts with an opportunity to identify *FDS* M&S requirements for use in naval platform survivability analysis. The utilisation of *FDS* also enabled further testing of the VSRS *Arion* model.

### Modelling assumptions

Due to the age of the platform upon which VSRS *Arion* was derived, material specifications were unavailable. Consequently, assumptions relating to material types and thicknesses were applied. Assumptions were also applied to the fire fuel load within the compartment in which the RPG detonated. Analysis of a platform during the design phase may require similar assumptions. For an existing, in-service platform, these details should be known. The assumptions applied in the case study were:

- material properties for platform structure were mild steel of 5 mm thickness;
- weapon impact location was predefined (see Section "Defining the scenario" *Defining the Scenario*);
- only one fire was considered (the one in the compartment in which the RPG detonated); and,
- the fire fuel load was a solid combustible material fire, known as a Class A fire.

Assumptions specific to the fire M&S tools were:

1. FSSIM:
  - the fire fuel load was uniformly distributed across the deck of the compartment in which the fire occurred;
  - the fire fuel load was 61.61 kgm^-2^; and
  - the heat release rate (HRR) was defined by a t-squared fire ramp with a fire growth coefficient of 0.015 kWs^-2^;
2. FDS:
  - the fire fuel load was concentrated at the centre of the compartment in which the fire occurred; and
  - the HRR was ramped from 0 to 600 kW over one minute and then remained at 600 kW.

The difference in assumptions between *FSSIM* and *FDS* related specifically to the use of the two M&S tools in the case study. *FSSIM* is a network model with the nodes representing compartments in VSR *Arion*. Due to a node having one set of parameters representing the fuel load for the compartment, the fuel load is considered as distributed throughout the compartment. Conversely, *FDS* is a CFD model where a compartment is meshed into many cells (the *FDS* user guide^23^ provides recommendations for mesh sizing). This means the fuel load can be distributed throughout the compartment. However, since *FDS* was not the primary fire model used in the case study, the nature of the fuel load distribution was not defined, and the fire ignition point was not identified. Consequently, for *FDS*, the fuel load was concentrated at the centre of the compartment. The physical size of the fire was defined by assuming a Class A fire, the size of the compartment, and ventilation, as supported by prior research^25–28^.

### Constraints

Constraints were imposed to reduce the complexity of the scenario to match the available resources (primarily, the availability of suitable analysts to perform the M&S required during the case study). The constraints were:

1. to limit susceptibility modelling and analysis (see below);
2. to only implement and model the functionality of platform systems required for the scenario;
3. to only implement crew, and model their behaviour, required for the scenario;
4. the effect of smoke on crew visibility and movement was not considered (smoke was only considered from the perspective of containment to stop it from spreading);
5. the effect of fire insulation was not considered; and,
6. the effect of flooding was not considered (however, due to the damage sustained during the scenario, this would be a consideration in a real situation).

Constraining the susceptibility analysis was a necessity due to the limited availability of resources (time, funding and analysts) in the available timeframe to perform the case study. Consequently, susceptibility was only considered in terms of recovering platform manoeuvring capability for the requirement of threat avoidance (an inner susceptibility layer of the Integrated Survivability Onion, Fig. 1). In normal circumstances, susceptibility analysis might consider acoustic and non-acoustic signatures, as well as tactical operations for threat avoidance, interception, and destruction.

### Verification and validation

Verification and validation (V&V) of the M&S tools is outside the scope of the case study. V&V is a future consideration. V&V for *FSSIM* and *FDS* are published in the literature^29–32^. *IRM* is verified and validated by NAVSEA Program Executive Office Program Management Services on an approximate per decade basis as the technology, applications, scientific data resources and computational capabilities evolve. *IRM*’s most recent V&V accreditation was issued in 2019.

V&V is an ongoing process and occurs using decommissioned naval platforms, onboard in-service platforms, within buildings approximating naval platforms, and using physical scale models approximating real world environments. For example, live explosive and fire and smoke trials, such as that performed on HMAS *Derwent*^19^, and crew movement trials^16,33^, have enabled the development and validation of relevant models for use in survivability analysis. Comparative and validation studies have also occurred for other M&S tools to be used in the Integrated Survivability analysis framework^24,33^. Due to security sensitivities, when V&V occurs for some tools (for example, *UVAM* and *IRM*) that analysis will not be available in the public domain.

## Defining the scenario

The scenario utilised the VSRS *Arion* model with configuration changes applied to technology associated with the fire main, and a change to deck configuration. The platform was exposed to a detonating RPG weapon threat, resulting in a fire. The RPG detonation caused a loss of functionality in two platform systems: one that supported platform survivability; and the other supporting a Command Aim to fulfil the mission objective. The survivability analysis was to identify a resilient platform configuration to achieve the Command Aim.

### Scenario description

VSRS *Arion*’s mission objective is to escort a high valued asset (HVA) transiting a narrow passage of water with dense levels of shipping. The Command Aim is to provide protection to the HVA by intercepting threats, thereby allowing the HVA to safely transit the narrow passage. Enemy combatants are known to operate in the area, taking advantage of the narrow passage and using the cover of other shipping. The enemy combatants utilise high-speed boats as launch platforms for shoulder-launched RPGs.

VSRS *Arion* detects a high-speed boat (the threat) approaching but, due to other shipping, is unable to safely engage the threat. Consequently, VSRS *Arion* is unable to destroy or dissuade the threat. The threat intercepts VSRS *Arion* and launches an RPG that impacts the starboard-stern of VSRS *Arion*.

**Fig. 5:**
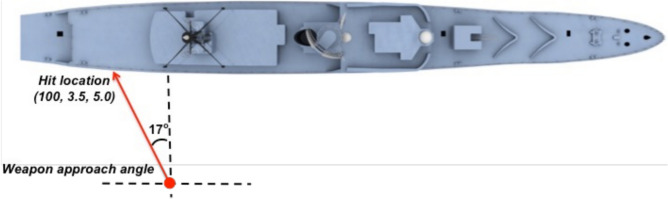
Plan view of VSRS *Arion* depicting weapon launch and platform hit locations (not to scale).

**Fig. 6:**
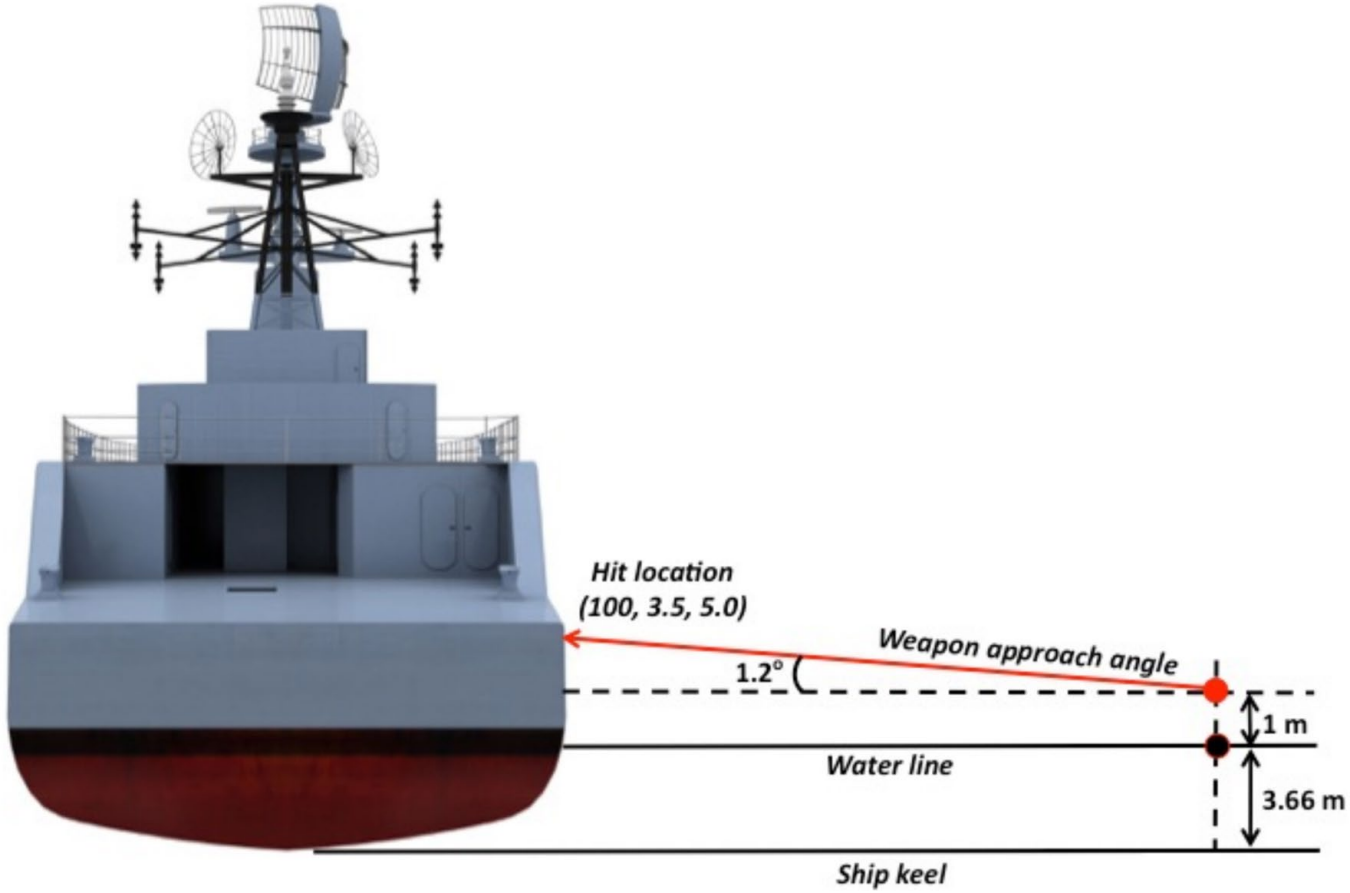
Stern view of VSRS *Arion* depicting weapon launch point and platform hit location (not to scale).

**Fig. 7:**
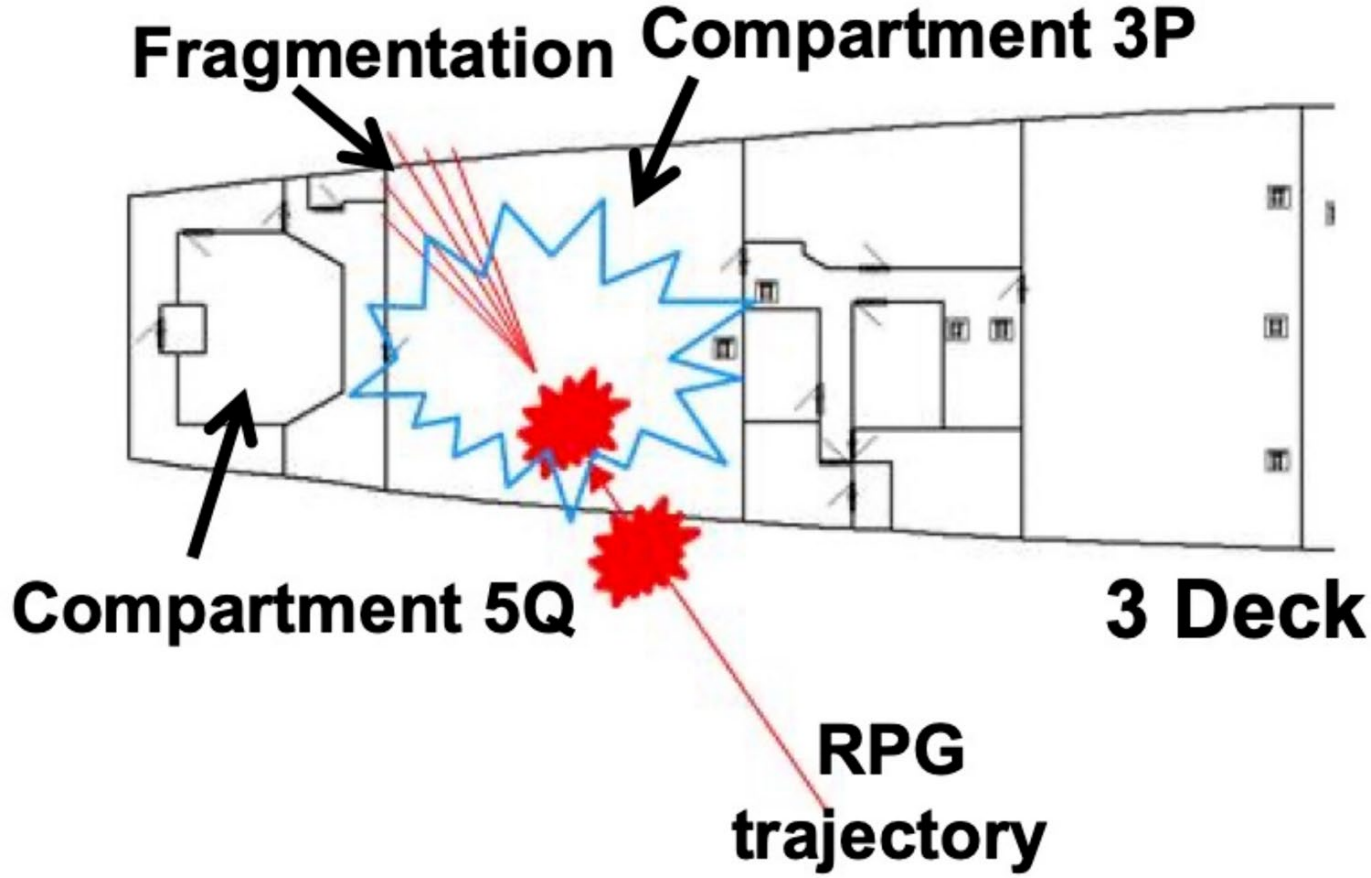
VSRS *Arion* stern depicting Compartment 3P and Compartment 5Q on 3 Deck and the location of the RPG detonation inboard of Compartment 3P.

The RPG consists of two explosive charges. The first charge acts as a hull penetrator to enable the second charge to detonate inboard of VSRS *Arion*. The location of detonation is in Compartment 3P, the stern crew quarters. Aft of Compartment 3P is Compartment 5Q, the steering equipment compartment. Figures 5 and 6 present detailed views of the scenario, depicting the weapon launch position, approach angles and the location of impact on VSRS *Arion*. The RPG impact location on VSRS *Arion* is (X, Y, Z) = (100, 3.5, 5.0) m measured from the global datum. Figure 7 depicts the stern general arrangement for 3 Deck of VSRS *Arion*, showing the location of the weapon detonation inboard of Compartment 3P.

Blast and fragmentation causes damage to the fire main in Compartment 3P. The result is a loss in water pressure to the fixed firefighting (sprinkler) system in Compartment 3P. Consequently, there is no firefighting capability within the compartment.

Fragmentation penetrates the bulkhead separating Compartment 3P and Compartment 5Q, causing damage to the steering equipment located within Compartment 5Q. The result is an immediate loss of platform manoeuvring capability. A loss of manoeuvrability means VSRS *Arion* is susceptible to further attack from enemy combatants loitering in the area of operations. Furthermore, without manoeuvrability, VSRS *Arion* is unable to achieve the Command Aim of protecting the HVA.

Hot fragmentation within Compartment 3P initiates a fire. The fire impedes the only access to Compartment 5Q. Without firefighting capability in Compartment 3P, the fire cannot be immediately extinguished or contained.

The Damage Control In-charge (IC) must identify a process to recover platform capability for the achievement of the Command Aim. This translates to a Command Priority of recovering the steering functionality that provides manoeuvring capability to the platform. To achieve that priority, the decision reasoning process is:

1. the steering equipment must be repaired;
2. to repair the steering equipment, crew need access to Compartment 5Q;
3. to access Compartment 5Q, the fire in Compartment 3P must be extinguished;
4. to extinguish the fire in Compartment 3P, recovery of the fixed firefighting system in Compartment 3P is required; and,
5. to recover the fixed firefighting system, the crew need to understand why the fixed firefighting system failed.

Consequently, the first objective to be achieved is to identify the location and cause of failure to the fixed firefighting system. To achieve the objective, the first action initiated by the Damage Control IC is to form up and task a Blanket Search Team to visually search the damaged compartments.

### VSRS *Arion* survivability objectives

VSRS *Arion* has a Command Aim of intercepting threats to ensure the HVA’s safe transit of the narrow passage of water. To maximise the probability of successful achievement of the Command Aim, VSRS *Arion* must restore manoeuvring capability within five minutes of weapon detonation. Between five and ten minutes, the probability of achieving the Command Aim diminishes. If manoeuvring capability is not recovered after ten minutes, the HVA becomes a combat casualty.

### VSRS *Arion* platform configurations

Four platform configurations were considered for assessment against the Command Aim. They were:

1. Baseline Configuration: the crew manually operate fire main valves to divert fire main water to supply the fixed firefighting system;
2. Remotely Operated Valve Configuration: valves to divert fire main water to supply the fixed firefighting system are remotely operated by the Damage Control IC;
3. Autonomic Valve Configuration: valves to divert fire main water to supply the fixed firefighting system are operated by a computer control system upon detecting and diagnosing a system fault; and,
4. Baseline Configuration with Access Hatch: a new access hatch is located on 2 Deck, giving direct access to Compartment 5Q. The crew manually operate fire main valves to divert fire main water to supply the fixed firefighting system.

**Fig. 8:**
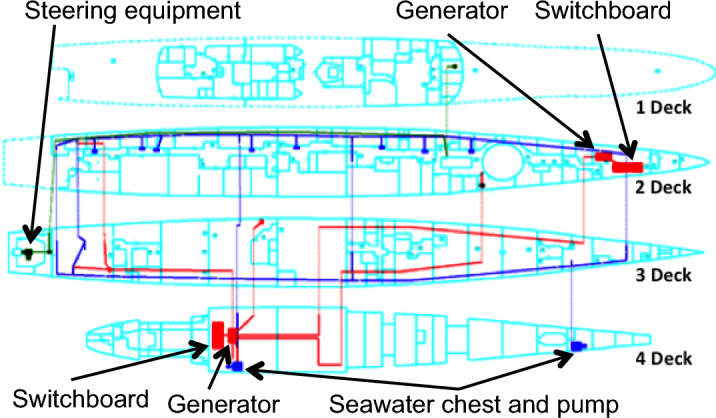
VSRS *Arion* fire main, fixed firefighting system and support system arrangement.

Shown in Fig. 8 is the fire main, the fixed firefighting system, and relevant support systems layout onboard VSRS *Arion*. Table 2 provides a key for each depicted system. The implemented systems were: the fire main and fixed firefighting (sprinkler) system; sea chests and pumps to distribute seawater through the fire main; and generators and power distribution switchboards to power the sensor system, fire main pumps, and activation of the sprinklers. Not shown in Fig. 8 are the sensors in Compartment 3P to detect the presence of fire and smoke. The sensors were analyst-defined attributes of the sprinkler system within *IRM*. The system layout shown in Fig. 8 did not change in the four platform configurations, only the implemented logic for valve activation and system operation changed (as modelled in *IRM*).

**Table 2:** Legend of systems presented in Fig. 8.

Symbol	Meaning
	Generator for power
	Switchboard for power distribution
	Seawater Chest and Pump for charging the fire main with seawater
	Power distribution
	Fire main and firefighting system
	Steering equipment (no functionality, except for crew interaction to enable repair)

**Fig. 9:**
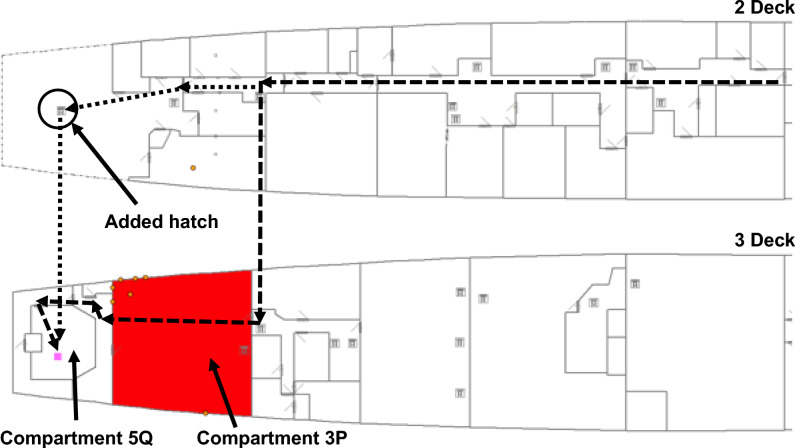
Two access routes to Compartment 5Q: the dashed line represents Configurations 1, 2 and 3; and the dotted line extends the dashed line to represent the access route for Configuration 4.

In Configurations 1, 2 and 3, there is only one access route to Compartment 5Q. That route is via Compartment 3P, which is obstructed by fire during the scenario. The dashed line in Fig. 9 represents this access route. In Configuration 4, a new hatch is included in the weather deck on 2 Deck connecting to Compartment 5Q on 3 Deck, thereby providing the crew with a secondary access route to Compartment 5Q. This is represented by the dotted line in Fig. 9, where the dotted line continues the path of the dashed line on 2 Deck to the location of the hatch. The solid shaded area represents the fire in Compartment 3P that must be extinguished prior to the Damage Control Team accessing Compartment 5Q, when following the route indicated by the dashed line.

In Configuration 1, the Baseline Configuration, the Blanket Search Team must identify the location and cause of failure to the fixed firefighting system. A Damage Control Team will then need to access valves in closest proximity to the ruptured fire main to effect repairs. Their repairs will enable re-direction of water flow around the ruptured fire main. If the sprinkler system cannot extinguish or contain the fire, Firefighting Teams will be required. The act of the Blanket Search Team identifying the damage, and the Damage Control Team transiting and performing repairs will result in delayed activation of the fixed firefighting system. Further delays will occur if there is need for Firefighting Teams to perform a manual attack on the fire using fire hoses.

In Configuration 2, remotely operated valves will enable crew to re-direct the fire main water flow without needing a Damage Control Team in position. The delay to activate the fixed firefighting system will be a result of the Blanket Search Team identifying the cause for the failure of the fixed firefighting system. Again, further delays may occur if there is a need for Firefighting Teams to perform a manual attack on the fire.

In Configuration 3, autonomic valves are remotely operated by a computer control system. Immediately upon sensors detecting a fault in the fire main, the computer diagnoses the problem and remedies the situation. This configuration does not require a Blanket Search Team to identify the cause of damage to the fixed firefighting system. This configuration also does not require a Damage Control Team to repair the fixed firefighting system. There may still be a need for Firefighting Teams if the fire is beyond the capability of the sprinkler system. However, it was hypothesised this configuration should have a faster response time (than Configuration 1 or Configuration 2) to recover functionality of the fixed firefighting system.

In Configuration 4, the addition of a hatch in 2 Deck, directly above Compartment 5Q, will avoid the need to traverse Compartment 3P (the location of the fire) and, consequently, will not require firefighting prior to compartment entry. This configuration retained the baseline fixed firefighting system configuration (as described for Configuration 1). This configuration does not preclude the need to suppress the fire. However, it was hypothesised there would be no requirement to extinguish the fire prior to recovering platform manoeuvrability.

## Modelling the scenario

A computer M&S framework to integrate the survivability modelling tools into a single capability is still in development^1,2^. Therefore, for this case study, survivability modelling was performed using the tools in isolation. The subsequent survivability analysis integrated the output to provide an assessment of the ability for the four platform configurations to support the Command Aim.

### Vulnerability: blast and fragmentation

Vulnerability blast and fragmentation modelling contributes to decision-making for maximising platform (including systems and crew) resilience to threat effects. This is achieved by, for example, informing zoning, separation and redundancy of systems onboard the platform. Vulnerability modelling of VSRS *Arion* utilised the bespoke analysis software, *UVAM*.

Modelling blast and fragmentation in *UVAM* utilises two distinct modules: *UVAM* PropertyEditor; and *UVAM* Solver. *UVAM* PropertyEditor enables the analyst to assign material data to the naval platform model. *UVAM* Solver performs blast calculations and ray tracing analysis for fragmentation to calculate *P*_*k*_ for individual systems and components.

#### *UVAM* data requirements

Data requirements for *UVAM* included elements of the VSRS *Arion* platform model and weapon threat. Specifically:

1. platform geometry data in the form of a three-dimensional CAD model detailing geometric properties of the platform and internal general arrangement;
2. material data, such as type, structural thickness, and geometric element type (that is, whether it is structural, or objects representing systems or components) for the respective geometry data;
3. weapon composition, characterised by yield and explosive type, casing and fragmentation details (material, number of generated fragments, and distribution pattern);
4. weapon impact velocity and fuse delay options; and,
5. capability afforded by system functionality and a connectivity model to assess survivability of system components. This functionality has yet to be implemented; however, *P*_*k*_ for systems and components can be calculated.

Developing the blast and fragmentation vulnerability model consisted of six steps:

1. import the VSRS *Arion* CAD model into the *UVAM* PropertyEditor;
2. assign material metadata to structural elements and component entities within the CAD model;
3. export the model from the *UVAM* PropertyEditor;
4. create a weapon definition file;
5. define the weapon aim point (impact location on VSRS *Arion*) and detonation point (Figs. 5 and 6); and,
6. import the platform model (as exported in step 3) and the weapon definition file into the *UVAM* Solver to calculate *P*_*k*_ for platform structure, systems and components.

#### Calculating the probability of kill

*UVAM P*_*k*_ calculations for each element type utilise empirical algorithms to calculate *P*_*k*_ as a consequence of blast and fragmentation. *P*_*k*_ is presented as a fraction of 1, where a value of 1 is 100% probability the element type was damaged such that it no longer provides functionality. Due to the security sensitivity of vulnerability algorithms, generic blast and fragmentation algorithms were used during this case study to represent VSRS *Arion*-weapon interaction.

The blast algorithm calculates *P*_*k*_ based on the proximity of the explosive charge to each element type within the vicinity of the blast. The generic, unclassified algorithm to calculate *P*_*k*_ due to blast, *Blast*.*P*_*k*_, used within *UVAM* (for this case study) is presented in Eq. (1).

1 $${Blast.P}_{k}=1-(\frac{{R}_{O}}{{R}_{D}})$$

where.

- *R*_*O*_ is the distance from the centre of detonation to the closest point of the object; and,
- *R*_*D*_ is the blast radius within which damage is likely to occur.

Equation (1) states that, as *R*_*O*_ decreases, *Blast*.*P*_*k*_ approaches 1 (that is, 100% *P*_*k*_). In this case study, *R*_*D*_ was 3.2 m (Table 1).

The fragmentation algorithm calculates the number of fragments that are likely to hit an object. This is calculated using a ray tracing technique and an analyst defined fragmentation dispersal pattern. The algorithm also calculates the residual velocity of fragments after impact with an object. The generic, unclassified algorithm used to calculate the residual velocity, *V*_*out*_, is presented in Eq. (2).

2 $${V}_{out}=0.5\times {V}_{in}$$

where:

*V*_*in*_ is the fragment velocity before impact with the object.

Equation (2) states the residual velocity of a fragment will be halved after impacting an object.

#### Blast and fragmentation vulnerability assessment

**Fig. 10:**
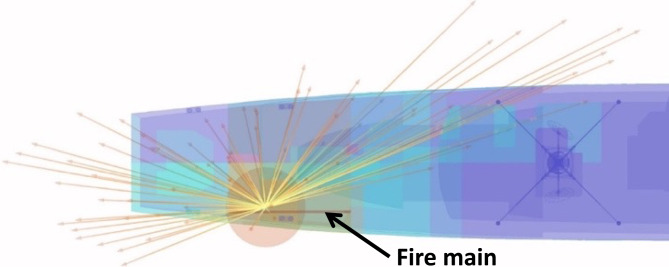
Top view of the weapon detonation inboard of Compartment 3P as modelled in *UVAM*.

**Fig. 11:**
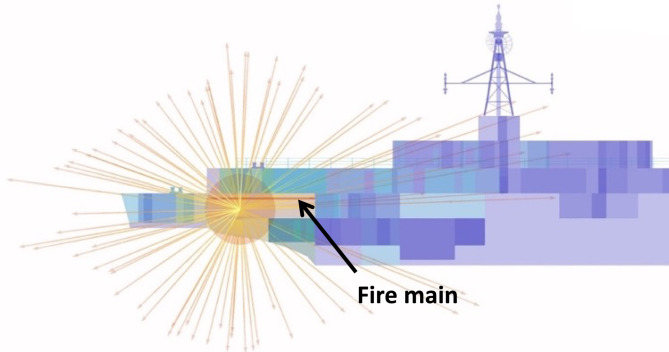
Starboard view of the weapon detonation inboard of Compartment 3P as modelled in *UVAM*.

**Fig. 12:**
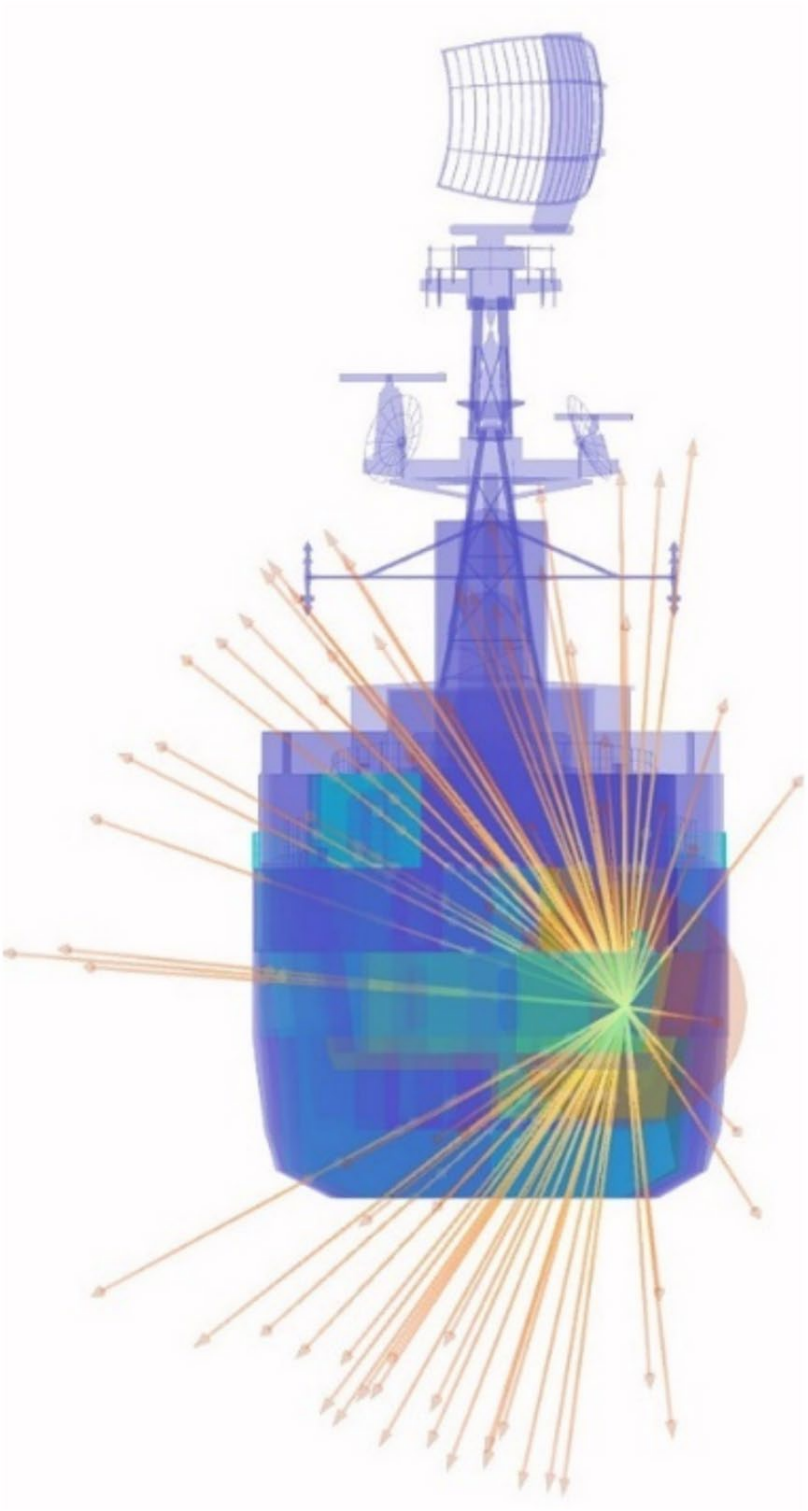
Stern view of the weapon detonation inboard of Compartment 3P as modelled in *UVAM*.

**Fig. 13:**
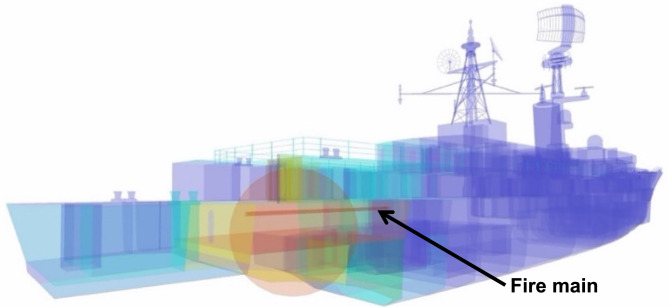
Starboard stern perspective view of the blast radius inboard of Compartment 3P as modelled in *UVAM*.

Blast and fragmentation vulnerability results are presented in Figs. 10, 11, 12, 13. Each figure presents a view of VSRS *Arion* showing the RPG detonation in Compartment 3P. The red circle represents the effective blast radius; and the vectors originating from the centre of the blast represent the fragmentation distribution ejected from the RPG detonation. Vectors in the current version of *UVAM* (at the time of performing the case study) only represent fragmentation direction, with the distribution pattern approximating that of an RPG. Objects affected by blast and fragmentation are shaded in different colours, representing the total *P*_*k*_ (combined *Blast*.*P*_*k*_ and *P*_*k*_ resulting from fragmentation impact) for each object. The colour codes are defined in Table 3.

**Table 3:**
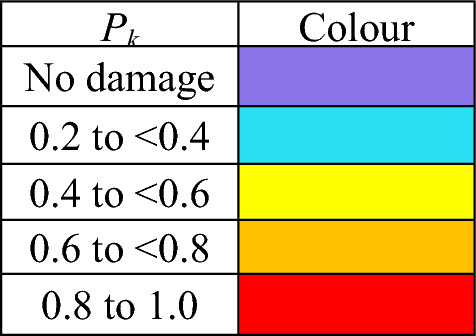
Colour codes representing *P*_*k*_ in Figs. 10, 11, 12, 13.

Visible in Figs. 10, 11 and 13 is the fire main within Compartment 3P, coloured red (indicating *P*_*k*_ of 80% to 100%). The fire main is also present in Fig. 12 but not visible due to the viewing angle.

*UVAM* generates *P*_*k*_ for individual objects within the damage zone. In Table 4, a list of structural objects and the fire main associated with Compartments 3P and 5Q are identified, along with *P*_*k*_ (resulting from the RPG detonation) for each object. In particular there was a single fragmentation impact to the fire main, part identifier CMP-00001621, located in Compartment 3P. Due to the fragment impact and the distance from the centre of the blast, *P*_*k*_ for the fire main was 0.819. Consequently, the fire main was likely ruptured, resulting in a loss of fire main water pressure. Without water pressure, the fire main is unable to supply the fixed firefighting system. Consequently, VSRS *Arion* lacked a firefighting capability in the zone encompassing Compartment 3P.

**Table 4:** Identification number and descriptions for several objects associated with Compartments 3P and 5Q.

Part Identifier	Part Description	Distance from blast (m)	Number of fragmentation hits	*P*_*k*_ (total)
STR-00001336	Hull located directly below Compartment 3P	2.37	16	0.550
STR-00000785	Deck of Compartment 3P	0.78	28	0.852
STR-00001505	Water-tight bulkhead between Compartment 3P and Compartment 5Q	2.95	12	0.437
STR-00001536	Water-tight door located on 2 Deck above Compartment 3P	1.70	0	0.462
CMP-00001621	Fire main located in Compartment 3P	0.95	1	0.819

Furthermore, the watertight bulkhead between Compartment 3P and Compartment 5Q, part identifier STR-00001505 in Table 4, received 12 fragment impacts. All these fragments penetrated the bulkhead. No components were modelled within Compartment 5Q in this case study. However, it is believed that, due to the number of penetrating fragments and the proximity to the centre of the blast, the steering equipment would have been damaged by the blast event. This was assumed to be true for the remainder of the study.

### Recoverability

Recoverability operations included two forms of firefighting (noting that firefighting and flood control are related to platform vulnerability to minimise damage to the platform). The first form of firefighting, using the fixed firefighting system (that is, sprinklers), was inoperative due to the damaged fire main. The sprinklers are mounted in the deckhead and they cannot be directed. Instead, they produce a wide dispersal spray pattern of water. The second form of firefighting was a “direct attack” performed by the Firefighting Teams using fire hoses that needed to be laid down and connected to the fire main. Firefighting with hoses enables the crew to manually direct water towards hotspots and the seat of the fire, and for boundary cooling (to slow the progress of heat transfer to other compartments). To understand crew behaviour and events during a damage scenario, Woolley et al.^2^ presented a summarised timeline of key events during the fire that occurred on HMAS *Westralia* in 1998^34^.

#### *IRM* recoverability model development

*IRM* model development included replicating the platform general arrangement within *IRM*, fitting out the required systems and crew, defining operational logic associated with systems and crew, and defining the damage model (such as, fire initiation). The platform systems include: inter-system dependencies; system failure and recoverability modes; crew skills necessary to operate and repair systems and equipment; and systems and mission fault trees for temporal capability assessment. Threat-based assessment (such as, output from *UVAM*) can be used to provide the initial damage input(s) for *IRM*. In this case study, the damage was defined manually within *IRM* using information calculated by *UVAM,* and the fuel load within Compartment 3P was defined during the scenario definition phase (see Section "Modelling assumptions" *Modelling Assumptions*).

**Fig. 14:**
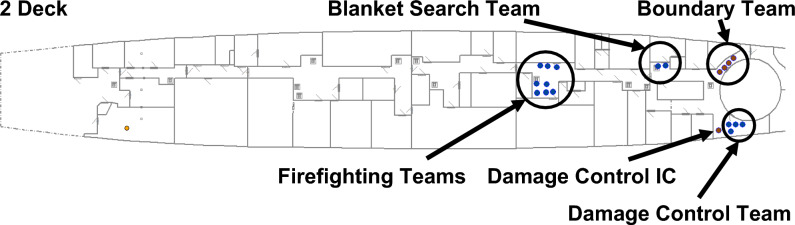
Crew locations on 2 Deck at the commencement of the scenario.

The only crew billets required for the scenario were: Damage Control IC, stationed in the Repair Base on 2 Deck, to manage the incident response; a Blanket Search Team of two crew; two Firefighting Teams of four crew each; a Boundary Team of four crew, to control the spread of smoke; and a Damage Control Team of four crew to repair and recover system functionality. Each team performed duties according to analyst defined activation logic associated with the respective systems. These can be equated to crew SOPs. Figure 14 presents the crew locations on 2 Deck at the commencement of the scenario.

#### *IRM* recoverability simulation

**Fig. 15:**
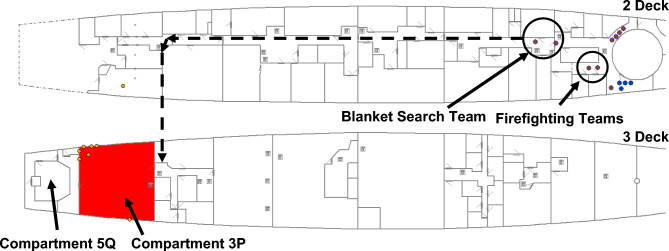
At 53 s after weapon detonation, the fire continues to expand in Compartment 3P, the Blanket Search team is moving to investigate the damage (following the dashed line), and Firefighting Teams have mustered in the Repair Base.

Recoverability simulation is exemplified using the Baseline Configuration and *IRM* screen captures at specific timestamps after RPG detonation. The state of VSRS *Arion* at 53 s after RPG detonation is presented in Fig. 15. Compartment 3P is shaded to represent the fire initiated by the RPG detonation. Firefighting Teams have mustered in the Repair Base; and the Blanket Search Team is following the dashed line in Fig. 15 to identify the damage.

Approximately 180 s after RPG detonation, the Blanket Search Team arrived at Compartment 3P and commenced the search for damage. They identified the presence of smoke and located the seat of the fire, reporting both to Damage Control IC. Damage Control IC then tasked the Boundary Team to establish smoke boundaries (to slow the progression of smoke into other compartments/zones, using smoke blankets), and tasked a Firefighting Team to extinguish the fire.

**Fig. 16:**
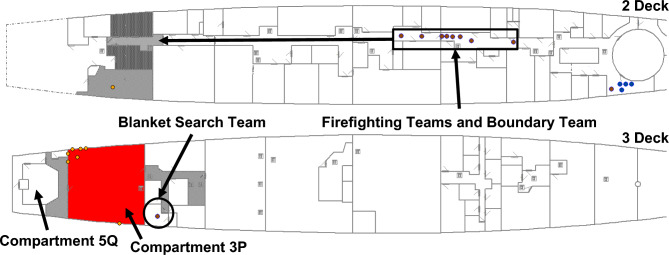
At 224 s after RPG detonation, the Blanket Search Team identified a ruptured fire main; the Firefighting and Boundary Teams are moving into position.

At 224 s after detonation, the Blanket Search Team identified the ruptured fire main and the resulting loss of functionality to the fixed firefighting system (sprinklers) in Compartment 3P. The loss of the fixed firefighting system significantly affected firefighting operations, necessitating a repair or a work around to be implemented. Damage Control IC is updated with the situation assessment. Damage Control IC then tasked the Damage Control Team to isolate the ruptured fire main and restore functionality to the fixed firefighting system. Concurrently, Firefighting Teams and the Boundary Team are moving into position. Crew locations at 224 s after RPG detonation are presented in Fig. 16. The grey shaded areas represent smoke dispersion to other compartments, with darker shades representing increased smoke density levels.

**Fig. 17:**
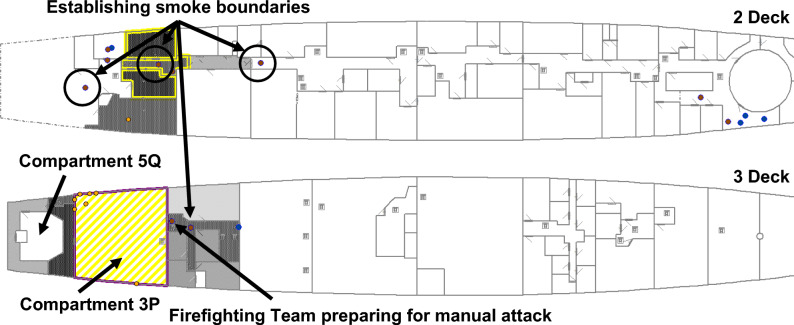
At 429 s after detonation, the ruptured fire main is isolated; the fixed firefighting system has activated (diagonal shading in Compartment 3P); and smoke boundaries established.

At 429 s after RPG detonation, the Boundary Team continued to establish smoke boundaries. The Damage Control Team isolated the ruptured section of the fire main and opened valves to restore fire main water pressure to recover functionality of the fixed firefighting system. The fixed firefighting system is activated, represented by diagonal shading in Compartment 3P shown in Fig. 17.

**Fig. 18:**
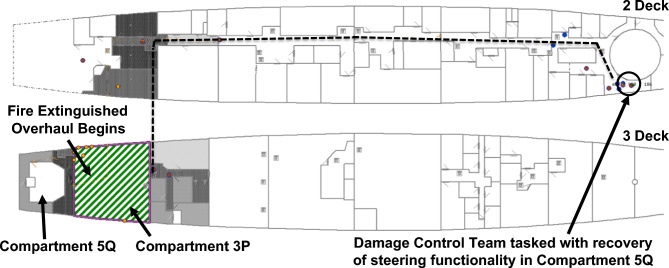
At 667 s after RPG detonation, the fire is extinguished and the Damage Control Team is tasked with repairing the steering equipment in Compartment 5Q. The dashed line depicts the route of the Damage Control Team.

After 481 s, manual firefighting, in the form of a direct attack on the fire performed by the Firefighting Teams, had commenced. The fire is extinguished 667 s after RPG detonation and the Damage Control Team is sent to Compartment 5Q with the task of recovering steering functionality. This is presented in Fig. 18. Finally, after 882 s, steering functionality is recovered and VSRS *Arion* has manoeuvring capability. In this configuration, it took the crew approximately 14 min to recover manoeuvring capability.

#### Recoverability results

Recoverability simulation output is presented in Table 5. The table presents the time to activate the fixed firefighting system, perform a direct attack on the fire, to extinguish the fire and recover steering functionality for the four platform configurations.

**Table 5:** Time (in seconds) to activate and perform firefighting, and to recover steering for the four platform configurations.

	Configuration			
	1. Baseline	2. Remotely operated valves	3. Autonomic valves	4. Baseline with access hatch
Activate fixed firefighting system	429	239	63	405
Perform manual attack	481	390	304	455
Fire extinguishment	663	423	336	637
Recover steering	882	654	576	168

**Fig. 19:**
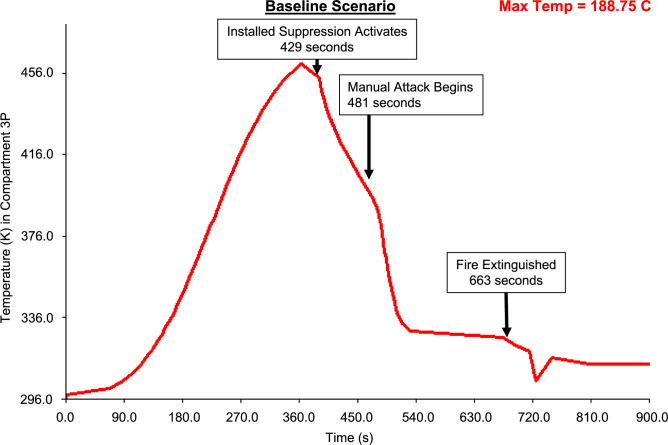
Fire temperature profile in Compartment 3P in response to the activation of the fixed firefighting system and manual firefighting for Configuration 1, baseline configuration.

**Fig. 20:**
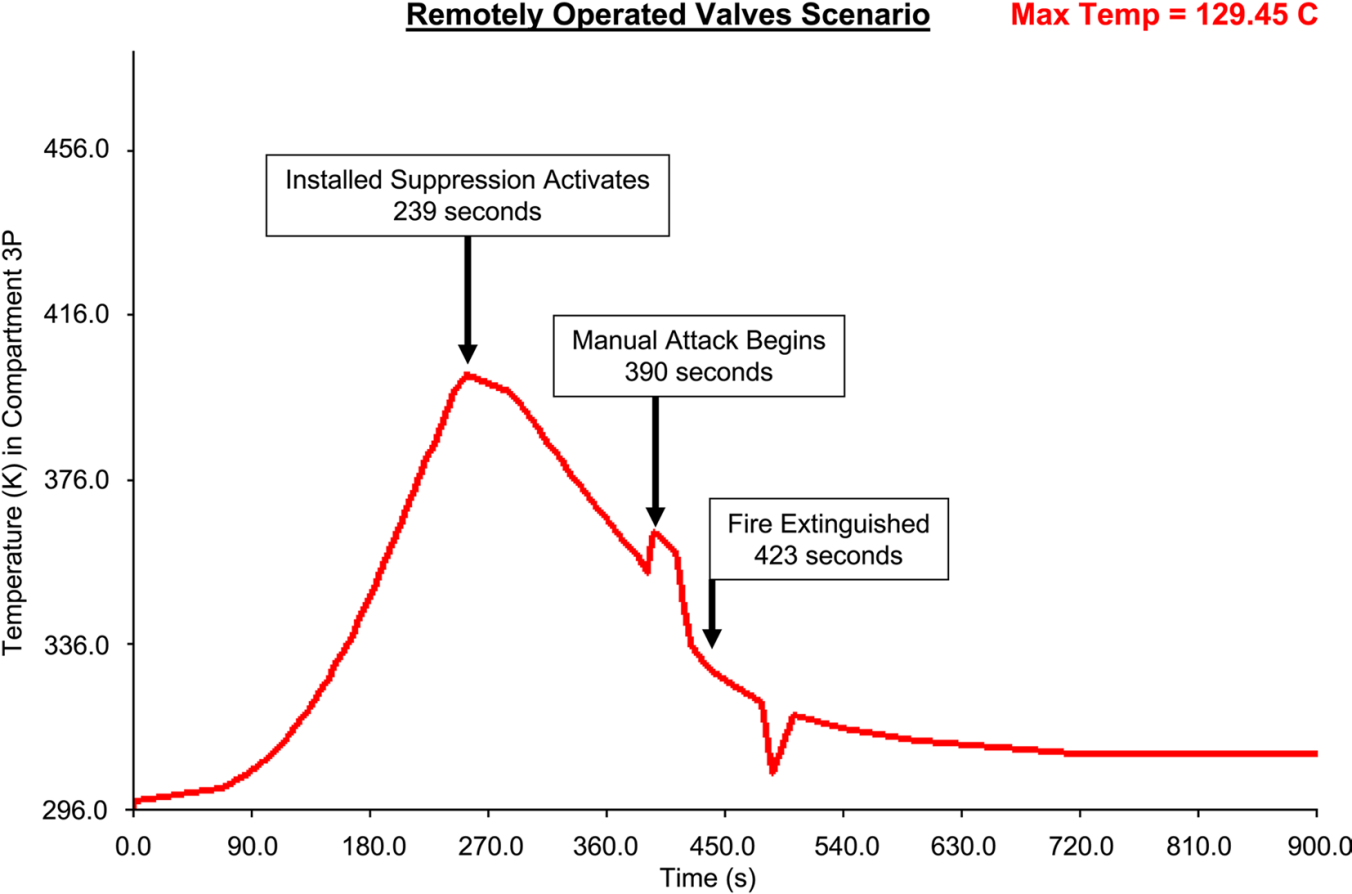
Fire temperature profile in Compartment 3P in response to the activation of the fixed firefighting system and manual firefighting for Configuration 2, remotely operated valve configuration.

**Fig. 21:**
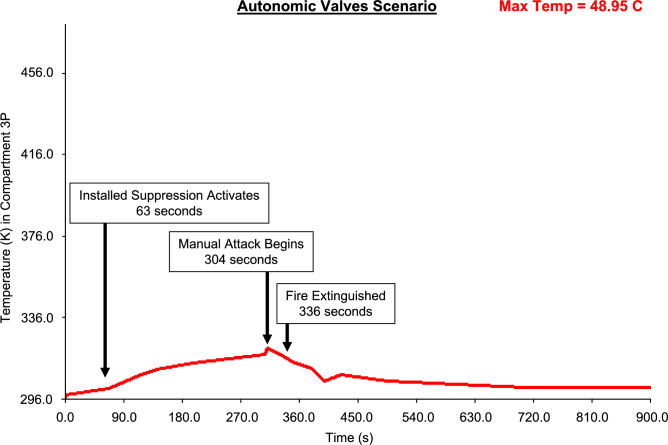
Fire temperature profile in Compartment 3P in response to the activation of the fixed firefighting system and manual firefighting for Configuration 3, autonomic valve configuration.

**Fig. 22:**
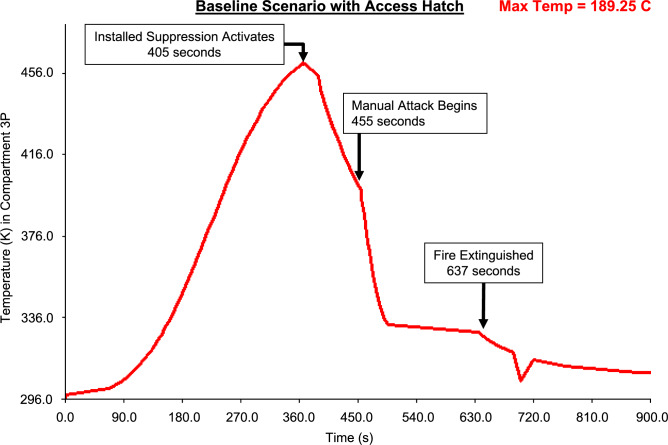
Fire temperature profile in Compartment 3P in response to the activation of the fixed firefighting system and manual firefighting for Configuration 4, baseline configuration with added access hatch.

Figures 19, 20, 21, 22 present fire temperature profiles in Compartment 3P for each of the four platform configurations. Highlighted in each profile is the time at which the fixed firefighting system activated, the time at which the Firefighting Teams commenced the direct attack, and the time when the fire was extinguished. In Configurations 1, 2 and 3, activation of the fixed firefighting system occurred at a progressively earlier time. Activation of the fixed firefighting system contained the fire to the compartment, giving the Firefighting Teams time to extinguish the fire by performing a direct attack on the seat of the fire. When the fire was extinguished, the Damage Control Team could enter the compartment and commence repairs on the steering equipment in Compartment 5Q. The temperature profile for Configuration 4 is comparatively similar to the temperature profile for Configuration 1. This is due to the utilisation of the same fixed firefighting system configuration (with variance due to the initial random seed). The fire temperature profiles for each of Figs. 19, 20, 21, 22 were generated using *FSSIM* in conjunction with *IRM*.

### Survivability analysis and discussion for the achievement of the command aim

In the scenario, VSRS *Arion* experienced an asymmetric threat in the form of a high-speed boat armed with a shoulder launched RPG. Platform susceptibility defences were compromised due to other shipping making it impossible to use self-defence weapon systems. Consequently, VSRS *Arion* sustained damage and lost the capability to achieve its Command Aim (protect the HVA by intercepting threats).

VSRS *Arion* became vulnerable to the threat when susceptibility defences were compromised. The consequent vulnerability events were:

1. RPG hull penetration (resulting in a hole in the hull);
2. RPG blast internal to Compartment 3P;
3. damage to the fire main;
4. initiation of a fire in Compartment 3P;
5. fragmentation penetrating the watertight bulkhead between Compartments 3P and 5Q; and,
6. damage to the steering equipment, resulting in a loss of platform manoeuvring capability.

**Fig. 23:**
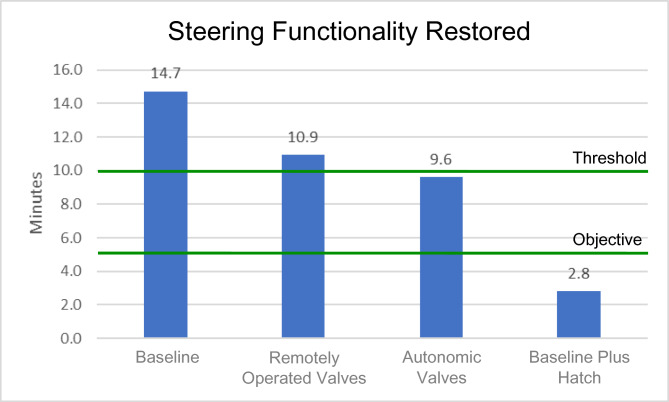
Time to restore steering functionality in the four different platform configurations.

The damaged fire main and the fire in Compartment 3P were the primary considerations after the RPG detonation. The fire was the only impediment to restoring platform capability for the achievement of the Command Aim. The time required in each of the four platform configurations to restore steering functionality (thereby restoring manoeuvring capability for the platform) is presented graphically in Fig. 23. The objective was restoration of manoeuvring capability within 5 min of RPG detonation. The threshold was 10 min, after which, the HVA would become a combat casualty. In Configurations 1 and 2, the threshold of 10 min was not achieved. In Configuration 3, restoration of steering functionality was achieved within 9 min and 36 s. The HVA would likely have become a combat casualty. Only Configuration 4 ensured the achievement of the Command Aim to protect the HVA.

The difference between Configuration 4 and Configurations 1, 2, and 3 is the inclusion of an access hatch. The hatch permitted direct access to Compartment 5Q, avoiding the need to extinguish the fire in Compartment 3P prior to accessing Compartment 5Q. Configurations 2 and 3 incorporated advanced technology into the platform system design. This technology was not sufficient to overcome the issue of a fire impeding access to a single point of entry compartment.

Four platform configurations were examined in this scenario. Two of those changes were technology related and one was a general arrangement configuration change. Other configuration changes could also be assessed (independently or working together). For example, consider the watertight bulkhead between Compartments 3P and 5Q, identified as STR-00001505 in Table 4. The bulkhead was 2.95 m from the centre of the blast and it received 12 fragmentation impacts. The resulting *P*_*k*_ of 0.437 (Table 4) indicates the bulkhead was damaged but, likely, continued to provide some of its design function. The bulkhead did not impede fragments from penetrating into Compartment 5Q. Utilising *UVAM*, assessment of protecting the bulkhead with armour plating can be performed to identify the effectiveness of stopping fragmentation penetrating Compartment 5Q.

The secondary vulnerability threat experienced by VSRS *Arion* was that of a fire in Compartment 3P. Detailed analysis of the rate of smoke spread, heat transfer, crew behaviour and the need for personal protective equipment (PPE), such as open circuit air breathing apparatus (OCCABA), was not examined in this case study. Gamble et al., using event trees, presented an example of the effects of some of these considerations during recoverability operations^24^. The inclusion of damage control and recoverability equipment to understand the effects on platform recoverability can be introduced when defining the *IRM* scenario.

There are multiple threat scenarios against which a platform will need to be assessed, and one configuration change suiting one scenario might not be acceptable for another scenario. Each scenario will need to be analysed, and configuration changes will need to be validated to ensure no adverse effects to crew safety or platform (and, therefore, mission) resilience. Capability trade-off analysis for each configuration change will need to occur to maximise mission success and minimise the platform’s risk of exposure to the threat environment. For example, upgrading to a laser weapon system (LaWS) for self-defence will require evaluating the availability of onboard space and weight margins; and it will also require an evaluation of power requirements and the ability of the platform to supply that requirement. Increasing hull plate thickness and/or the inclusion of armour plating will also require evaluation of operational performance (due to the extra weight), speed, weight margins, and stability; as well as an evaluation of acoustic and non-acoustic signatures.

For the scenario in this case study, other platform considerations to examine for improved platform resilience to achieve the Command Aim, might include:

- upgrading the self-defence system(s) to one that enables precision targeting and guidance (such as LaWS or high velocity projectile) to engage asymmetric threats in a threat environment that precludes the use of (current) conventional weapon systems;
- increasing hull thickness to impede threats;
- increasing thickness of the watertight bulkhead between Compartment 3P and Compartment 5Q to impede fragmentation;
- fitting armour plating to the watertight bulkhead between Compartment 3P and Compartment 5Q, to impede fragmentation; or,
- any combination of the aforementioned, including a hatch above Compartment 5Q and the utilisation of smart valve technology to maintain water pressure in the fire main.

Detailed analysis of operational tactics to avoid or intercept the threat will also be required.

### Outcome and recommendation for VSRS *Arion*

Platform criticality identified in this scenario is attributed to a single access route to effect repairs. Therefore, the recommendation to ensure VSRS *Arion*’s achievement of the Command Aim in this scenario is:

- 1. incorporate a new access hatch to improve accessibility to critical equipment in Compartment 5Q.

Even though technology insertion did not result in achievement of the Command Aim for this scenario, technology insertion did improve platform recoverability times. Consequently, other combinations of technology insertion may improve survivability in this and other scenarios. Therefore, further recommendations include:

- 2. analyse system criticality, redundancy and accessibility in all single point of entry compartments;
- 3. examine the incorporation of armour plating around critical equipment in Compartment 5Q;
- 4. analyse smart technology associated with ensuring the safety and operations of all critical systems for improved platform survivability;
- 5. analyse technology upgrades to the platform’s self-defence system; and.
- 6. examine operational tactics to avoid, engage and/or destroy the threat.

The case study only examined one Command Aim for one mission objective and one threat scenario. Further analysis will be required to ensure perceived upgrades do not adversely affect other aspects of the platform capability requirement or achievement of the mission requirement. Platform survivability analysis will also need to assess mission resilience against other mission requirements the platform will be expected to achieve and the threat environment to which it will be exposed. This will form part of the capability trade-off analysis to identify the optimum mix of platform mission related capabilities and survivability control measures that will maximise mission resilience against the threat environment.

## Discussion

Platform survivability analysis has traditionally been performed with respect to the individual domains of susceptibility, vulnerability and recoverability. The M&S analysis documented in relation to the case study generated output by performing vulnerability and recoverability analysis in isolation. Specifically:

- the vulnerability analysis demonstrated the ability to model blast and fragmentation effects, highlighting damage to the fire main resulting from the RPG detonation;
- fire modelling demonstrated the growth of the fire exceeded the ability of the firefighting capability (crew and systems) to suppress and extinguish in a suitable time frame; and
- recoverability modelling demonstrated the ability to recover system functionality using SOPs.

The case study was constrained to one generic platform and one damage incident. This was a deliberate constraint imposed to achieve the first objective of the case study: demonstrate the value of integrating the output from the individual survivability domains of susceptibility, vulnerability and recoverability. However, while this constraint may seem limiting, the concepts and methodology used in the case study can be generalised for application in assessing naval platform integrated survivability in any wartime or peacetime scenario. This was demonstrated in fulfilment of the case study’s second and third objectives. They were to: identify modelling and data requirements to perform integrated survivability analysis; and develop a workflow to perform future integrated survivability analysis (suitable for any given naval platform in any given survivability scenario). Achievement of these objectives is discussed.

### Objective 1: Demonstrating the concept of integrated survivability

The M&S of the scenario in this case study for the individual domains of vulnerability and recoverability identified damage sustained by the platform resulting from the RPG detonation; demonstrated the progression of fire and smoke; identified the time to extinguish the fire; and identified the time required for the crew to repair damage and recover platform capability. The output from the vulnerability analysis demonstrated the effect on defining subsequent elements of the scenario. That is, the blast damage to the fire main affected the operation of the fixed firefighting system and the ability to extinguish the fire. The blast also damaged the steering equipment. The location of the damage and ongoing secondary events (the fire and smoke) defined the priority actions to be performed by the crew to effect repairs and restore platform capability for the achievement of the Command Aim. Individual scenarios may be defined for blast analysis, for fire and smoke analysis, and for recoverability analysis; however, combining all three, as in this case study, demonstrates the consequences of one survivability domain affecting the conditions of other survivability domains.

Platform configuration changes can be examined within each of the susceptibility, vulnerability and recoverability domains to demonstrate the effect the changes have on a particular aspect of survivability. For example, configuration changes to the fixed firefighting system showed that different configurations resulted in changes to fire extinguishment times. However, for this scenario, configuration changes to the fixed firefighting system were not effective to enable the crew to restore platform capability within the required time frame.

The blast and fragmentation vulnerability analysis identified the connecting hatch between Compartments 3P and 5Q was not likely to have been damaged such that it did not impede access. However, the crew would not have that information until the fire had been extinguished and access to Compartment 3P had been achieved. When exposed to such a situation, the crew might consider cutting through 2 Deck to either gain direct access to Compartment 5Q; or to provide firefighting access, from above, for Compartment 3P. However, this presents risk due to cabling and pipe work behind the area through which they are cutting.

The combined outputs from the vulnerability, fire and smoke, and recoverability analyses showed that a general arrangement configuration change (the addition of a hatch) might be an effective solution to improve platform survivability. The integrated analysis identified an option that may not have been considered from the outset for platform design. The inclusion of the hatch may prove more effective than solutions identified by performing susceptibility, vulnerability and recoverability analysis in isolation. The nature of the scenario and the need to analyse mission resilience could only be achieved by taking all three survivability domains into consideration.

The case study was constrained in the susceptibility domain and further work will be required. A broader Integrated Survivability analysis study is planned and will include aspects of susceptibility analysis for an airborne weapon threat and the ability of the platform to avoid and/or destroy that threat. However, even though the case study had limitations, the M&S and subsequent analysis satisfied the first objective of demonstrating the concept of integrated analysis for platform survivability with respect to a Command Aim.

### Objective 2: M&S and data requirements

The second objective, to identify M&S and data requirements, revealed a need to clearly define the analysis objectives and scenario prior to commencing any M&S. The nature of the case study allowed for errors to occur; and for the discovery of requirements to perform future Integrated Survivability M&S. The primary requirements discovered during the case study related to the platform CAD model; a need to define platform material properties; and a need to define crew SOPs. Importantly, though, there is a need to clearly define the scenario, including goals to be achieved from the analysis and objectives to be achieved within the scenario. These requirements are discussed in the following sub-sections.

#### Defining analysis goals and scenario objectives

Clearly and concisely defined goals and objectives facilitate the creation of a suitable scenario to test hypotheses and answer analysis questions. In turn, this will facilitate defining platform mission objectives to be achieved within the scenario; defining analysis metrics; and identification of suitable M&S tools to perform the M&S and subsequent analysis. The M&S tools will then dictate modelling and data requirements. A formalised, clear and concise definition of analysis goals and scenario objectives will give confidence in the output and ensure avoidance of “ad hoc” decision-making during the M&S and analysis process.

#### The platform CAD model

The VSRS *Arion* CAD model was originally constructed such that many major features were produced as a single CAD object. This meant it was not possible to deconstruct the platform CAD model to allow for examination of individual zones or compartments. For example, decks within the VSRS *Arion* CAD model were each created as one object and individual compartments could not be separated from that object. Consequently, this may result in computer processing overheads when modelling a scenario (for example, lack of processing memory and/or increased run time). Therefore, the VSRS *Arion* platform model will need to be re-examined to ensure CAD objects within the model can easily be de-constructed when needed. For future analyses, using platforms other than VSRS *Arion*, there will be a similar need to ensure platform models have separate CAD objects representing individual aspects of the platform.

#### Platform specification

The platform specification (such as material properties and fire fuel loads) must remain consistent across all M&S tools used during the analysis. For existing platforms, platform specification should be known; however, during the early design phase for any given platform, some of these properties may need to be assumed. In this case study, using VSRS *Arion*, not all the properties for the platform upon which VSRS *Arion* was derived were known and, therefore, were assumed.

#### Standard operating procedures

SOPs for crew behaviour must be defined during the scenario specification phase. If not, the crew may exhibit unexpected behaviour and there will be time consuming delays to debug the models. Liu and Woolley, and Woolley and Liu presented a methodology to define crew behaviour that might be suitable when codifying crew actions in recoverability modelling software^35,36^. Other techniques^37^, that may be more suitable, are being examined.

### Objective 3: methodology workflow to perform integrated survivability analysis

The third objective for the case study, the development of a methodology workflow to perform platform Integrated Survivability analysis, was also achieved. This workflow may be used to assess naval platform integrated survivability during platform acquisition, mission planning, and upgrades to the fleet-in-being.

**Fig. 24:**
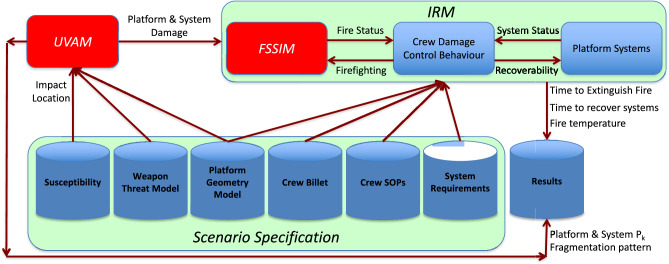
Simplified model and data connectivity within the case study. Information from *UVAM* was manually built into the *IRM* model.

Figure 24 present a simplified representation of information transport between the M&S software used in the case study. Information contained within “Susceptibility”, “Weapon Threat Model”, “Platform Geometry Model”, “Crew Billet”, “Crew SOPs”, and “System Requirements” were defined during the case study definition phase. *UVAM* outputs the damage caused by the weapon detonation and this was used to manually define the damaged systems within *IRM*. *IRM* utilised the same “Platform Geometry Model” and utilised analyst defined “Crew Billet”, “Crew SOPs” and “System Requirement” information to populate the platform model. The models within *UVAM* and *IRM*, for this case study, were developed concurrently. Within *IRM*, when the simulation commences, relevant system, fire and crew updates occur concurrently. Actions within *IRM* occur when specific logic requirements are met. This logic can be written in plain English as, for example, “if the fire is extinguished then the crew performs recoverability operations”.

**Fig. 25:**
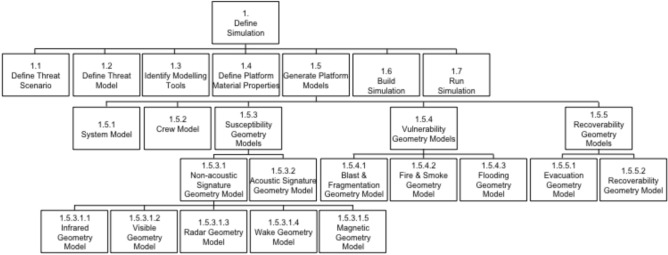
Hierarchy workflow to define and perform Integrated Survivability analysis.

Using details presented in Section "Defining the case study" *Defining the Case Study*, Section "Defining the scenario" *Defining the Scenario*, Section "Modelling the scenario" *Modelling the Scenario* and Fig. 24, a hierarchy workflow was developed. An extract of the workflow is shown in Fig. 25. The same workflow is presented in tabular form in Table 6, which includes a description for each step in the workflow. The workflow identifies the need to define and understand the threat scenario to which the platform will be exposed. This can be a combat threat (that is, a weapon) or a non-combat threat (for example, a collision with another platform, grounding, or a fire). There will be a need to identify the M&S tools to analyse the scenario and the subsequent need to generate platform models in the respective formats required by the M&S tools. The workflow will require refinement and validation.

**Table 6:** Tabulated workflow to define and perform Integrated Survivability analysis.

Step ID	Title	Description
1	Define Simulation	Specify the scenario and M&S requirements to perform platform Integrated Survivability analysis
1.1	Define Threat Scenario	Define the requirements for the survivability scenario
1.2	Define Threat Model	Define the threat(s) to which the platform will be exposed
1.3	Identify Modelling Tools	Based on details defined in ‘1.1 Define Threat Scenario’ and ‘1.2 Define Threat Model’, identify M&S and analysis tools that will be utilised to examine the scenario
1.4	Define Platform Material Properties	Define material properties (for example, construction material, bulkhead thickness) for the platform
1.5	Generate Platform Models	Identify M&S requirements for the selected analysis tools and generate platform, system and crew models in the respective formats
1.5.1	System Model	Identify and generate system models required in the scenario
1.5.2	Crew Model	Define crewing requirements and generate crew behaviour models
1.5.3	Susceptibility Geometry Model	Identify geometry format and generate geometry models required for the susceptibility M&S tools
1.5.3.1	Non-acoustic Signature Geometry Model	Identify geometry format required for non-acoustic signature M&S analysis and generate models
1.5.3.2	Acoustic Signature Geometry Model	Identify geometry format required for acoustic signature M&S analysis and generate models
1.5.4	Vulnerability Geometry Models	Identify the geometry format required for vulnerability modelling and generate models
1.5.4.1	Blast & Fragmentation Geometry Model	Identify geometry format required for blast and fragmentation M&S analysis; generate models
1.5.4.2	Fire & Smoke Geometry Model	Identify geometry format required for fire and smoke M&S analysis; generate models
1.5.4.3	Flooding Geometry Model	Identify geometry format for flooding M&S analysis; generate models
1.5.5	Recoverability Geometry Models	Identify geometry format required for recoverability M&S analysis; generate models
1.6	Build Simulation	Build the requisite simulations within modelling tools contributing to the analysis for the scenario
1.7	Run Simulation	Run the simulation

Table 6 can be considered a “check-list” for the process to follow when defining the scenario. Noting, not all items may be required for any given scenario. Due to space limitations, details have been omitted from Fig. 25 and Table 6. For example, expansion of “1.1 Define Threat Scenario” provides a detailed list of considerations that may need to be included during the threat encounter. An extract of those considerations is presented in the workflow of Table 7. Three ellipses in the table indicate further details have been omitted (for conservation of space).

**Table 7:** Workflow for “1.1 Define Threat Scenario”.

Step ID	Title	Description
1.1	Define Threat Scenario	To define the requirements for the survivability scenario
1.1.1	Scenario Description	Detailed description of the scenario, including threat(s), assumptions and constraints
1.1.2	Mission Requirement	Specification of the platform’s mission objectives
1.1.2.1	Platform Capability Requirement	Identification of platform capability requirements to achieve mission objectives
…	…	…
1.1.2.2	Scenario Success Criteria	Define the success criteria for achievement of the objectives for the mission requirement
1.1.2.3	Scenario Measurement Criteria	Define measures to assess the scenario
…	…	…
1.1.3	Platform Survivability Capability	Identify survivability capability available to the platform for the specified scenario
…	…	…
1.1.4	Threat Types	Define the types of threats to which the platform will be exposed during the scenario
1.1.4.1	Combat Threat	Define the combat threats to which the platform will be exposed during the scenario
…	…	…
1.1.4.2	Non-combat Threats	Define threats that do not originated from combat
…	…	…
1.1.4.3	Secondary Effects	Primary damage event(s) may initiate secondary events
…	…	…
1.1.5	Environmental Conditions	Define environmental conditions to which the platform and threat are exposed during the scenario
…	…	…

Some details that appear in the workflow may be generated by physics-based modelling or defined prior to performing analysis. For example, when defining the threat scenario, trajectory and the impact location of a “fly-in” (or, “swim-in”) weapon threat may be performed using specific M&S tools to simulate the threat behaviour in response to platform behaviour (such as, manoeuvring to avoid the threat). Alternatively, these criteria may be specified by the analyst, thereby avoiding (for whatever reason) simulating the behaviour of the threat on approach to the platform.

The entire workflow constitutes the design threat scenario presented by Woolley and Whitehouse^1^. The considerations for designing the platform-threat interaction (where the threat might be a combat threat, or a peace-time threat) suitable to perform Integrated Survivability analysis are explicitly and tacitly incorporated into the workflow as represented by Fig. 25, and Tables 6 and 7.

### Identified M&S issues

The objectives of the case study contributed to understanding the requirements of performing Integrated Survivability analysis. The majority of the M&S tools utilised in the case study contributed directly to the analysis of the scenario and achieving the objective of demonstrating integrated analysis for platform survivability. However, the case study was also an opportunity to utilise software that had not previously been examined for naval platform survivability analysis. In particular, the authors had not previously examined *FDS*. In this case study, *FDS* was used to identify modelling requirements and further test the utilisation of the VSRS *Arion* CAD model.

**Fig. 26:**
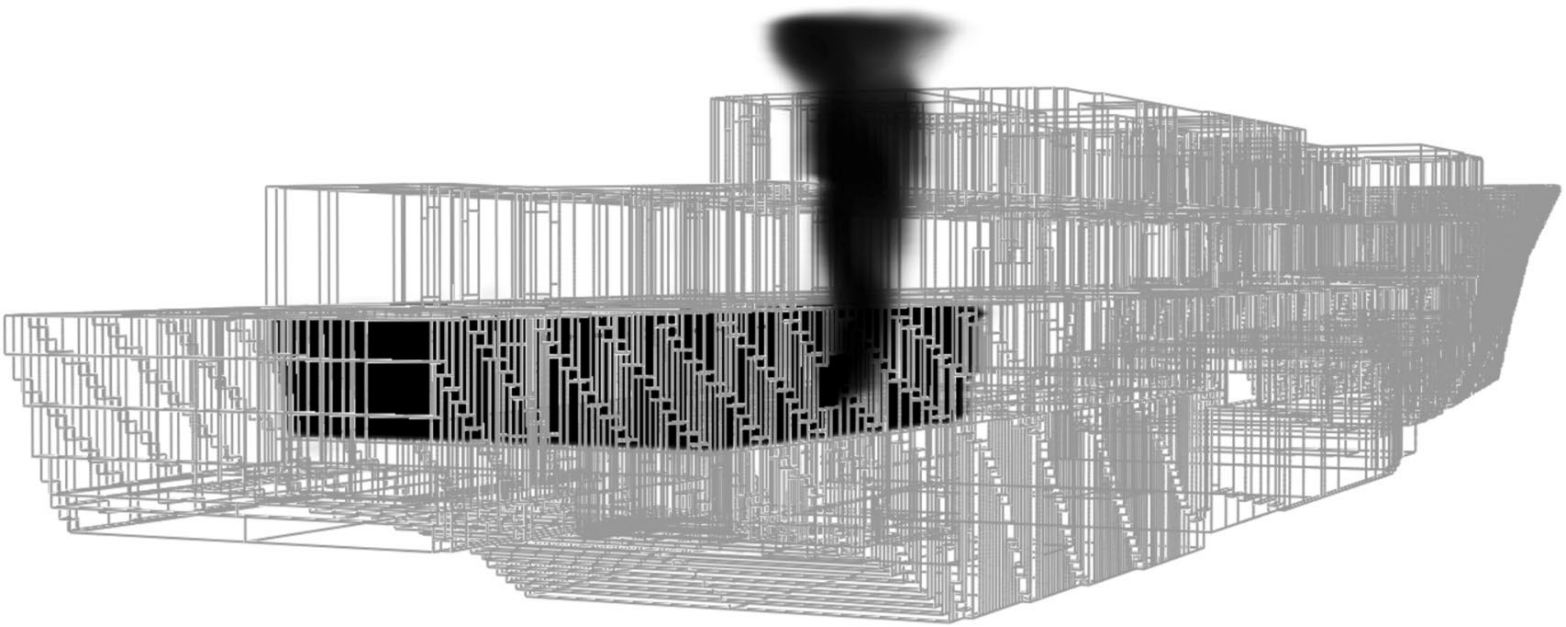
*FDS* wireframe model depicting venting from the weapon entry hole.

**Fig. 27:**
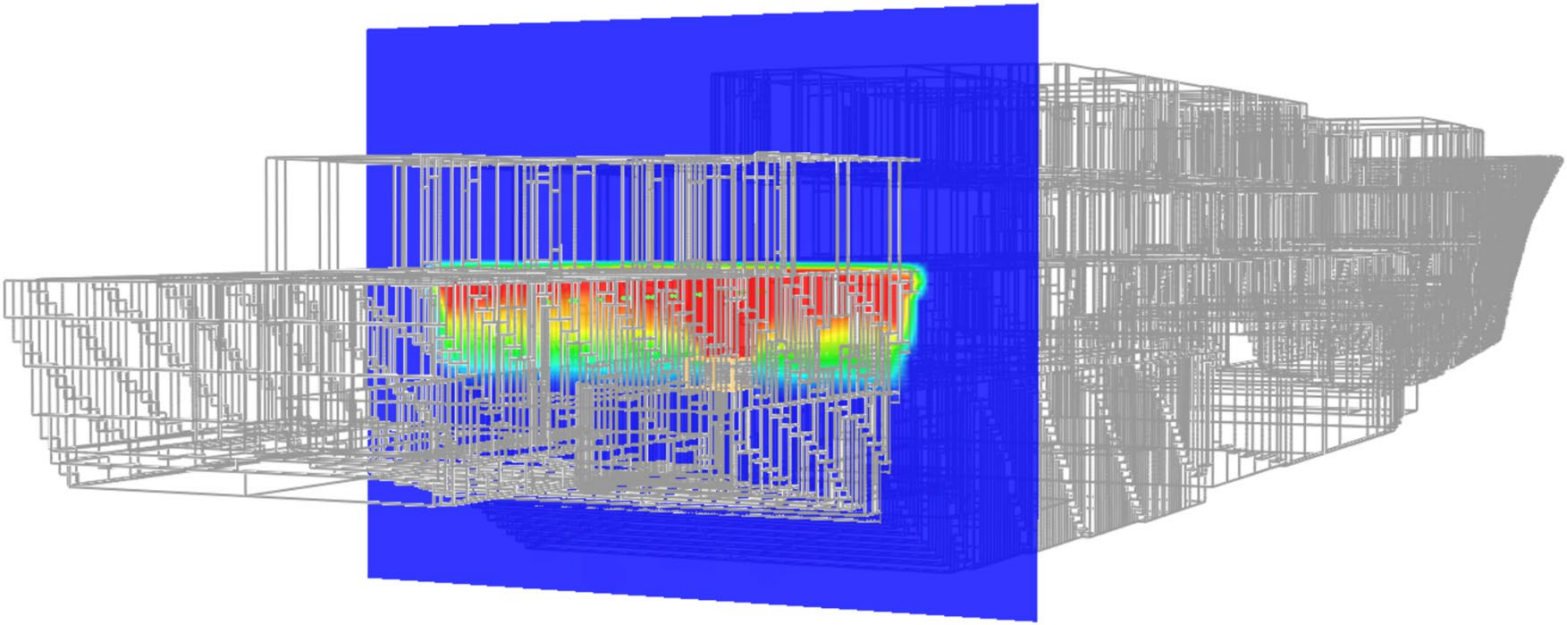
*FDS* fire temperature profile at a specific timestamp during the *FDS* fire simulation. The temperature ranges from blue (ambient temperature) through to red (approximately, 170^o^ C at this specific timestamp).

**Fig. 28:**
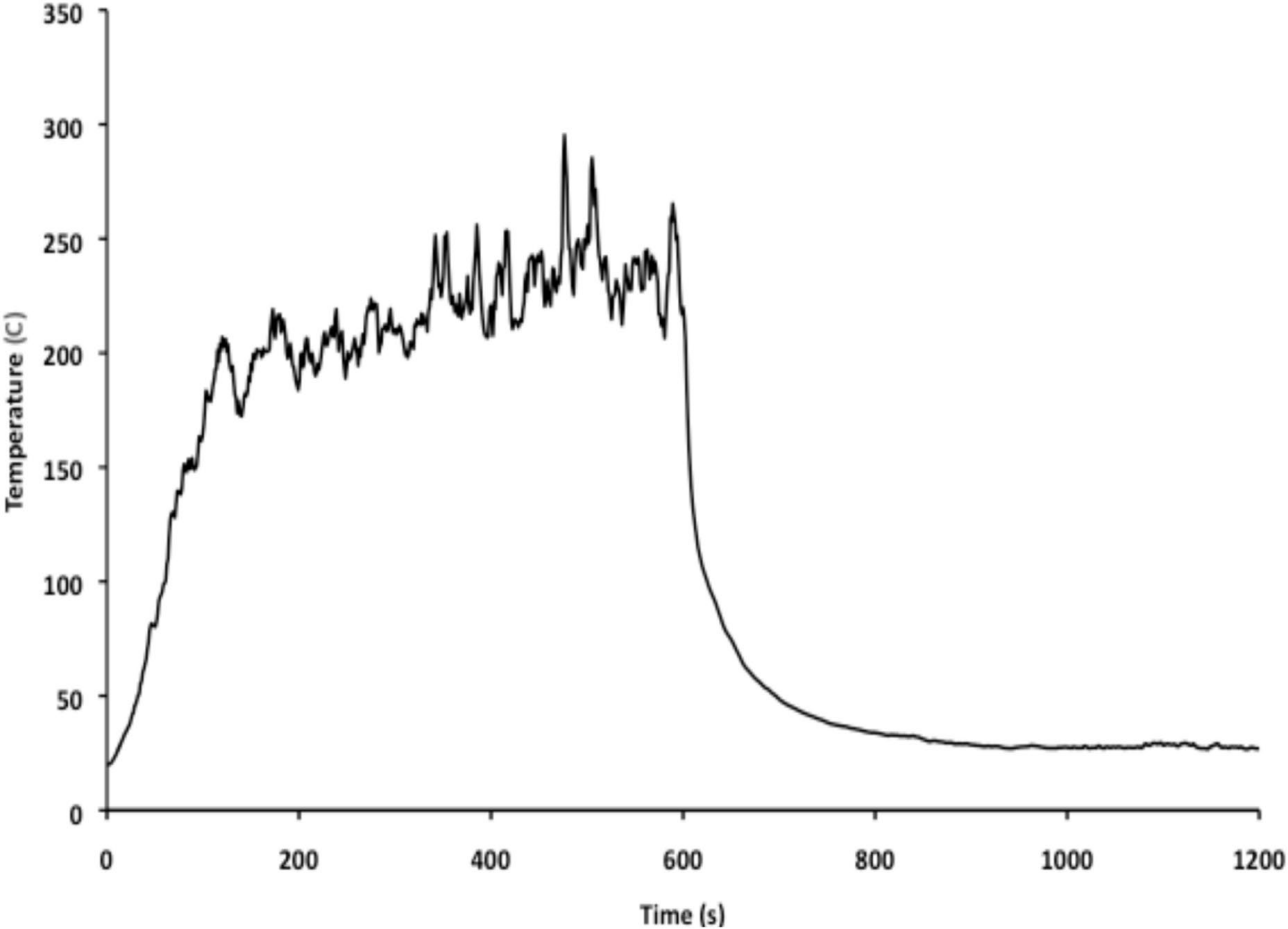
Fire temperature curve in Compartment 3P as modelled in *FDS*.

The use of *FDS* did identify an issue with the VSRS *Arion* CAD model. Preprocessing for *FDS* was performed using *PyroSim*^38^ to facilitate the assignment of material properties and for the removal of unnecessary CAD surfaces to reduce computational overhead. However, due to the method of constructing the original VSRS *Arion* CAD model, each deck was modelled as one continuous object. Consequently, the decks could not be manipulated within *PyroSim* without affecting the area of interest (that is, Compartments 3P and 5Q). Fortunately, this was overcome within *FDS* when defining the mesh size that divided the platform structure into cells to model the progression of fire and smoke. Figure 26 presents a wireframe model of VSRS *Arion* within *FDS*, showing smoke venting from the weapon entry hole. The dark shaded rectangular area is Compartment 3P. Figure 27 presents a cut plane temperature profile at a specific timestamp during the fire simulation, while Fig. 28 presents the fire temperature curve from *FDS* for the full duration of the fire. The fire was modelled in *FDS* as if no firefighting had occurred. It is possible to model the effects of firefighting; however, this requires understanding of crew behaviour and the time required to perform specific actions in the lead up to enable firefighting. This leads into the second issue identified with analysing the scenario—understanding crew behaviour and the time to perform specific actions.

Fleet standard timings specify time frames in which the crew must achieve their desired outcomes to ensure the safety of their platform and themselves. These timings could be incorporated directly into *FDS* to define when specific actions should occur. However, this would require alignment with crew actions in the recoverability M&S tools, such as *IRM*. There is also no connectivity between *FDS* and *IRM* to synchronise the simulation. *IRM* does have connectivity with *FSSIM* but aligning the *IRM* pedestrian movement timing with fleet standard timings would be an involved process. Also, validation of the fleet standard timings will be required. Detailed understanding of crew behaviour needs to be developed and codified such that it can be implemented in *IRM*. Other work by Woolley et al.^2^ is developing capability to model crew behaviour during naval platform recoverability operations for analysing the effect of crew actions on platform survivability.

### Integrated survivability analysis for platform acquisition versus the fleet-in-being

The presented case study is akin to decision-making associated with the survivability analysis during the design phase for capability acquisition or during refit to an in-service platform. However, similar analysis can be performed for evidenced based decision-making during the process to ensure seaworthiness^39^ and readiness of a platform prior to deployment. The risk management process for seaworthiness is a holistic examination platform defects and deficiencies. Seaworthiness requires that those defects be rectified or suitable contingencies are in place for the defects to be safely managed. Similarly, readiness is a holistic examination of the capability requirement for any given deployment and quantifying mission success. Both of these processes have a requirement for a survivable platform. Integrated Survivability analysis provides a scientific, evidenced based solution contributing to risk identification and rectification for seaworthiness, and to capability analysis for the mission profile of a deployment.

## Ongoing work

This case study utilised M&S tools used in isolation, with the analysis integrating the output from each tool. Ideally, susceptibility analysis would examine the threat-platform interaction and provide threat impact information (for example, impact location and velocity) to the blast vulnerability analysis. Likewise, output from the susceptibility modelling (for example, residual fuel load for a fly-in weapon threat) and from blast vulnerability modelling would be automatically input into fire and smoke vulnerability analysis and recoverability analysis to initiate respective M&S in those domains. Platform status would then be updated on a continuous basis to understand the effects on platform susceptibility, vulnerability, recoverability and mission capability for the duration of the scenario. There is, currently, no M&S framework to enable communication between the respective M&S tools. Also, there is no capability to model an ongoing scenario. However, such a framework has been proposed^1^ and is currently in development with a scaled down framework known as *NavDIRecT*^2^. The intended development path for *NavDIRecT* is for it to evolve into the *Integrated Ship Survivability Assessment Capability* (*ISSAC*)^1,2^.

*ISSAC* is a proposed M&S framework utilising a simulation games engine to provide software links for the automation of simulating a threat encounter. The case study presented in this paper has contributed to further the scientific development of *ISSAC*. The workflow (as exemplified in Tables 6 and 7) will contribute to the refinement of the functional requirements and identify connectivity between models within the framework.

## Conclusion

An Integrated Survivability case study was performed using a selection of available M&S tools to analyse a threat scenario involving a representative naval platform. The study demonstrated the capability of the M&S tools contributing to mission resilience analysis of four platform configurations to support the achievement of a Command Aim.

The analysis highlighted the ability to examine the naval platform survivability trade-space by comparing platform survivability technology insertion and platform general arrangement configuration. The naval platform was exposed to a weapon threat that damaged platform capability required to protect an HVA. The ongoing threat environment meant it was imperative the platform recover the capability within a specific timeframe (akin to fleet standard timings) to ensure the safety of the HVA. The threat initiated a fire onboard the platform. The fire impeded the ability of the recoverability teams to restore platform capability prior to the threat returning in the specified time frame. This was not possible using the proposed platform survivability technology examined in the scenario. Instead, changes to platform general arrangement enabled crew to effect repairs in the required time to maximise survivability of the mission and against the ongoing threat environment.

An outcome from the case study identified the need for consideration of the scenario description, threat specification and platform configuration specification. Consequently, a detailed methodology workflow was generated to support the development of future platform survivability scenarios. This work flow represents the design threat scenario to enable development of appropriate metrics to measure platform survivability and mission resilience, and ensure all M&S tools used in such studies are utilising equivalent platform and threat specifications. In an Integrated Survivability analysis framework, a considered scenario description and the application of available M&S tools can contribute to supporting resilient, agile, seaworthy, mission ready platforms.

## Data Availability

Data sets generated during the study are available from the corresponding author on reasonable request.

## Abbreviations

*Blast*.*P*_*k*_: Probability of kill due to blast
m: Metre
*P*_*k*_: Probability of kill
*R*_*D*_: Blast radius within which damage is likely to occur
*R*_*O*_: Distance from the centre of detonation to the closest point of an object
*V*_*out*_: Fragment residual velocity (after impact with an object)
*V*_*in*_: Fragment velocity (before impact with an object)
CAD: Computer aided design
CFD: Computational fluid dynamics
DSTG: Defence Science and Technology Group
FDS: Fire Dynamics Simulator
FSSIM: Fire and Smoke Simulator
HMX: High melting explosive
HRR: Heat release rate
HVA: High valued asset
IC: In-charge
IRM: Integrated Recoverability Model
ISSAC: Integrated Ship Survivability Assessment Capability
LaWS: Laser weapon system
M&S: Modelling and simulation
NavDIRecT: Naval Damage Incident Recoverability Toolset
NAVSEA: Naval Sea Systems Command
NIST: National Institute of Standards and Technology
OCCABA: Open circuit air breathing apparatus
OPNAVINST: Office of the Chief of Naval Operations Instruction
PPE: Personal protective equipment
RAN: Royal Australian Navy
RPG: Rocket-propelled grenade
SSEP: Ship Survivability Enhancement Program
SOP: Standard operating procedure
TSS: Total Ship Survivability
UVAM: Unified Vulnerability Assessment Model
V&V: Verification and validation
VSRS: Virtual Survivability Research Ship
VuLCAn: Vulnerability Lethality Capability Analysis
